# Multiplex dynamic networks in the newborn brain disclose latent links with neurobehavioral phenotypes

**DOI:** 10.1002/hbm.26610

**Published:** 2024-02-05

**Authors:** Mohammad Al‐Sa'd, Sampsa Vanhatalo, Anton Tokariev

**Affiliations:** ^1^ BABA Center, Pediatric Research Center, Department of Clinical Neurophysiology, Children's Hospital, HUS imaging, HUS Diagnostic Center University of Helsinki and Helsinki University Hospital Helsinki Finland; ^2^ Department of Physiology University of Helsinki Helsinki Finland; ^3^ Faculty of Information Technology and Communication Sciences Tampere University Tampere Finland

**Keywords:** brain networks, dynamic functional connectivity, electroencephalogram, multiplex networks, neurodevelopment

## Abstract

The higher brain functions arise from coordinated neural activity between distinct brain regions, but the spatial, temporal, and spectral complexity of these functional connectivity networks (FCNs) has challenged the identification of correlates with neurobehavioral phenotypes. Characterizing behavioral correlates of early life FCNs is important to understand the activity dependent emergence of neurodevelopmental performance and for improving health outcomes. Here, we develop an analysis pipeline for identifying multiplex dynamic FCNs that combine spectral and spatiotemporal characteristics of the newborn cortical activity. This data‐driven approach automatically uncovers latent networks that show robust neurobehavioral correlations and consistent effects by *in utero* drug exposure. Altogether, the proposed pipeline provides a robust end‐to‐end solution for an objective assessment and quantitation of neurobehaviorally meaningful network constellations in the highly dynamic cortical functions.

## Graphical abstract

Investigating cortico‐cortical network relationship to neurobehavior in newborns is important to understand activity‐dependent neurodevelopment; however, identifying subnetworks that correlate with phenotypes is challenging due to the high complexity of neuronal interactions. We developed a method for analyzing electroencephalography connectivity networks that uncovers links to neurobehavioral traits and evaluates early‐life development.

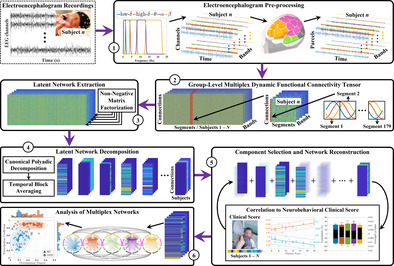

## 1 INTRODUCTION

The higher brain functions are supported by the rapid dynamics in spatially distributed networks with synchronized neuronal activity (Bullmore & Sporns, 2009; Hutchison et al., 2013). These neuronal interactions, or functional connectivity networks (FCNs) (Bullmore & Sporns, 2009; Lurie et al., 2020), can be estimated from the statistical relationships in signals that originate from neural activity, such as electroencephalography (EEG) (Abbas et al., 2021; Uchitel et al., 2022) or magnetoencephalography (MEG) (Stam et al., 2007; Tewarie et al., 2019). Despite the widely acknowledged success of network neuroscience in explaining brain functions from molecular to systems level (Bassett & Sporns, 2017), it has been particularly challenging to establish functional network correlates to various neurobehavioral phenotypes, such as diagnostic entities or neuropsychological performance (Tokariev et al., 2021; Yrjölä et al., 2021; Yrjölä et al., 2022). This challenge is likely linked to the natural richness, or technically speaking the high‐dimensional complexity in the FCNs spontaneous occurrings (Tokariev et al., 2021; Yrjölä et al., 2021; Yrjölä et al., 2022).

Studies on the functional connectivity are usually focused on measuring time‐averaged neuronal coupling at specific oscillatory frequencies; however, neuronal activity is known to exhibit multiplex network dynamics (Bassett & Sporns, 2017; Zhu, Liu, Mathiak, et al., 2020; Zhu, Liu, Ye, et al., 2020) characterized by rapid changes at sub‐second time scales (Bassett & Sporns, 2017; Haartsen et al., 2020; Panwar et al., 2021), as well as coincident interactions at a wide range of oscillatory frequencies (Bassett & Sporns, 2017; Haartsen et al., 2020; Westende et al., 2020). Recently, several systematic approaches of multiplex network analysis were proposed to accommodate coexisting networks operating at different oscillatory frequencies (Kivelä et al., 2014), and/or to measure the network dynamics seen as time‐varying changes in the network properties (Du et al., 2018; Haartsen et al., 2020). These approaches are typically compressing or factorizing the FCNs high‐dimensional representation to obtain stable latent dynamics, but they generally lead to an obscure access to the global FCN structures found in a clinical group. For instance, correlating raw FCNs (Shellhaas et al., 2022) with neurobehavioral phenotypes can lead to unreliable and/or unrepeatable findings due to the spontaneous random coupling naturally found in resting state neural activities and the intersubject acute differences within a clinical group. Moreover, factorizing the group‐level FCNs into components reduces intersubject variability (Mehrkanoon et al., 2021; Zhu, Liu, Mathiak, et al., 2020), but this process breaks the FCN's intrinsic patterns to become fragments scattered across the multiple components (Mahyari et al., 2017), which leads to depreciating their correlates with the neurobehavioral phenotypes and hinders interpretation. A recently proposed alternative reduces the high dimensionality of FCNs through a multivariate decomposition to produce a low‐dimensional representation of highly resolved time‐frequency interdependencies (Mehrkanoon et al., 2014). Despite its apparent analytical success in decomposition, such approaches suffer from lacking interpretability backward, that is, they are not based on frequency‐ or coupling mode‐related networks that would allow intuitive interpretation in the context of prior infant studies (Omidvarnia et al., 2015; Tokariev et al., 2019; Tokariev et al., 2021; Tokariev et al., 2022; Yrjölä et al., 2021; Yrjölä et al., 2022). One plausible solution to this challenge is to extract latent networks from the cohort‐level FCNs by finding low‐rank approximations (Mahyari et al., 2017) to suppress random and subject‐specific fluctuations before the decomposition into subnetworks of lower entropy (Du et al., 2018; Mahyari et al., 2017). Besides, the time‐varying behavior of these multifrequency, or multiplex, networks can be further incorporated to form multiplex dynamic FCNs (mdFCN); they extend the static connectivity definition and describe associations among brain regions in short‐time windows and multiple frequency bands (Haartsen et al., 2020).

**FIGURE 1:**
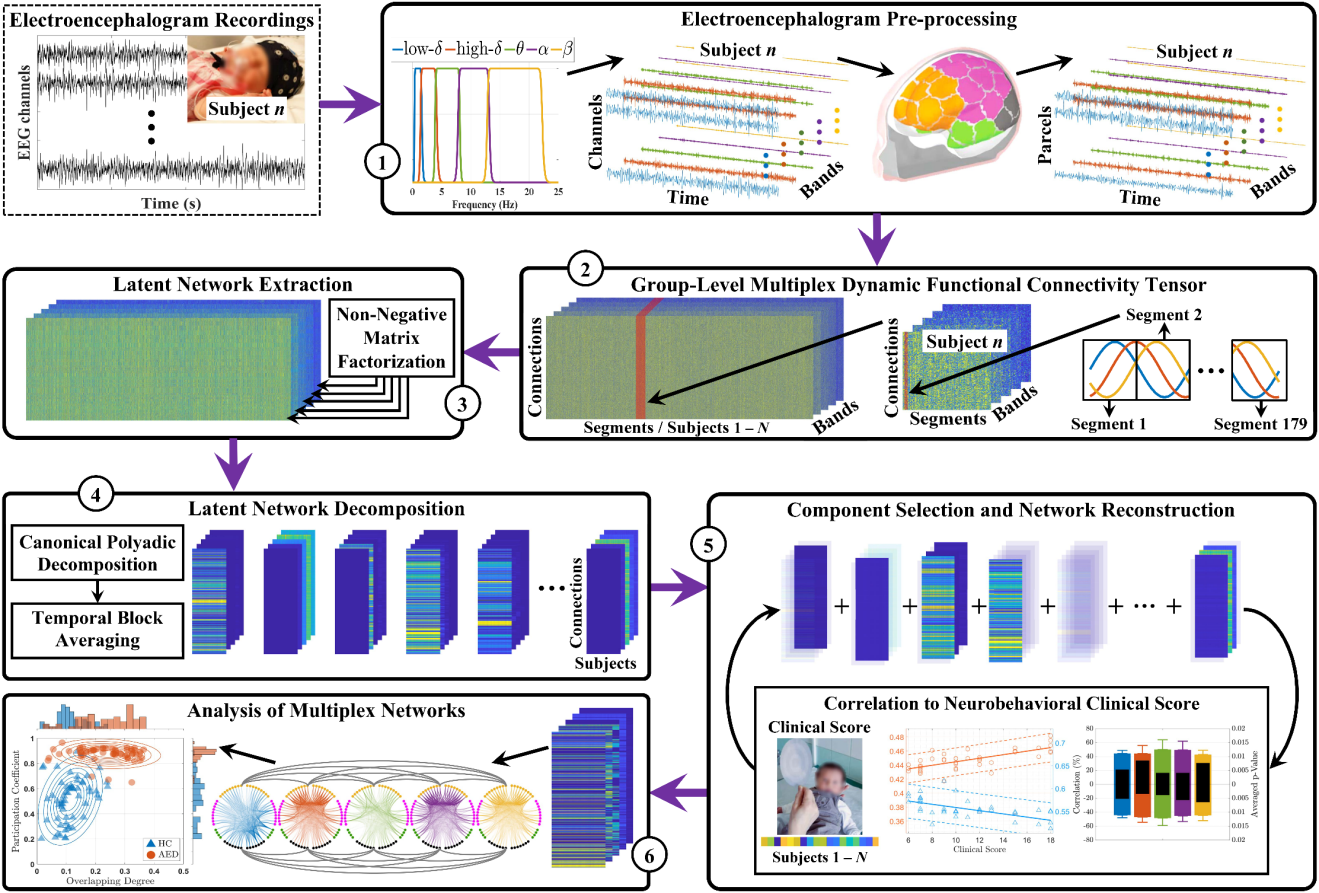
The study overview. The multichannel newborn electroencephalography (EEG) recordings were submitted to the multiplex dynamic functional connectivity network (mdFCN) pipeline that proceeds through the following stages: (1) preprocess the EEG into cortical source signals at distinct frequency bands; (2) compute the connectivity matrices using phase–phase synchronization in 2 s sliding windows in each infant and frequency band, which are then accumulated to a group‐level tensor; (3) extract the latent representation from the group‐level mdFCN via matrix factorization; (4) decompose the latent network into components via tensor rank decomposition and block‐average the temporal patterns to describe connectivity across subjects; (5) select a subset of the decomposed components for reconstruction and retain connections with significant correlations to the neurobehavioral phenotypes; and (6) analyze the spatial structures in the multiplex networks and compare their correlations to the neurobehavioral phenotypes in different clinical groups.

There is a particular scientific and medical need to better understand the early life correlations of FCNs and neurobehavior. Most cortical FCNs are known to organize during late pregnancy and early postnatal life in an activity‐dependent manner (Oldham & Fornito, 2019), making early brain development exquisitely sensitive to a range of early life adversities, such as exposure to drug treatments (Tokariev et al., 2021; Tokariev et al., 2022; Videman et al., 2016), prematurity (Omidvarnia et al., 2015; Tokariev et al., 2018; Tokariev et al., 2019; Uchitel et al., 2022; Westende et al., 2020; Yrjölä et al., 2021; Yrjölä et al., 2022), or perinatal adversities (Abbas et al., 2021; Rocca et al., 2020). In this work, we propose a novel mdFCN analysis pipeline for objectively uncovering latent network structures from the spontaneous cortical activity in newborn infants (Figure 1 shows a graphical overview of the study). The present study builds on the idea that clinically significant network characteristics are hidden in the high variance that characterizes FCNs between subjects, brain areas, frequencies, and time. In essence, we hypothesize that these FCN characteristics can be extracted as latent properties in the high‐level noise, and they can be uncovered by the developed mdFCN analysis pipeline. Additionally, the newly constructed networks would then link to the neurobehavioral phenotypes.

## 2 MATERIALS AND METHODS

### 2.1 Subjects

We used previously collected cohorts of neonatal multichannel scalp EEG recordings from altogether *N* = 120 infants recorded at the New Children's Hospital, Helsinki University Hospital, Finland: a healthy control (HC) group with *N* = 68 infants, and a group of *N* = 52 infants exposed *in utero* to maternal treatment with antiepileptic drugs (AEDs) (Tokariev et al., 2021; Videman et al., 2016). Infants in both groups were born full‐term with no significant difference in gestational age; HC at 40.3 ± 1.1 weeks and AED at 40.1 ± 1.4 weeks (average ± standard deviation).

### 2.2 EEG recordings

The multichannel scalp EEG recordings were collected for the HC and AED groups during daytime sleep using a NicOne EEG amplifier (Cardinal Healthcare/Natus, USA) with a sampling frequency of 250 or 500 Hz. The neonatal Waveguard caps had 20–32 sintered Ag/AgCl electrodes (ANT‐Neuro, Germany) placed according to the 10–20 international placement standard. The EEG signals were captured near term age (HC group: 42.2 ± 0.9 weeks; AED group: 42.1 ± 0.9 weeks) for about an hour that included both cardinal sleep states, active (AS), and quiet sleep (QS). Most subjects had clean enough EEG data for both sleep states (*N* = 53/46 for the HC/AED group), whereas others had only one state (Tokariev et al., 2019; Tokariev et al., 2021; Tokariev et al., 2022). The infants' sleep state was identified, as described in detail before (André et al., 2010), visually by the standard combination of electrophysiological and behavioral measures. In brief, newborn EEG exhibits continuous fluctuations during AS with irregular respiration and occasional eye movements, and in contrast, it is inherently discontinuous during QS and accompanies distinct regular breathing.

### 2.3 Neurobehavioral assessment

The neonatal period assessment (short‐term) was conducted at term‐equivalent age by the Hammersmith Neonatal Neurological Examination (HNNE) (Spittle et al., 2016; Videman et al., 2016). The HNNE is comprised of a large set of clinically observed or tested neurobehavioral items in different domains, but they were combined into two low‐dimensional components (C1 and C2) that correlate with later motor and cognitive/social performances (Tokariev et al., 2018; Tokariev et al., 2021; Yrjölä et al., 2021). Moreover, the long‐term development was evaluated at 24.3 ± 0.3 months by a psychologist following the Bayley Scales of Infant and Toddler Development (Albers & Grieve, 2007; Videman et al., 2016). These evaluations were combined into five developmental subdomains: cognition, language comprehension, language production, fine motor skills, and gross motor skills. The short‐ and long‐term clinical scores were not available for all the infants in the dataset. Specifically, the HC/AED group had 52/52 and 58/40 infants with short‐ and long‐term scores, respectively, with a similar number of AS/QS recordings (HC: 47/51 and 52/57, AED: 51/47 and 39/37 for the short‐ and long‐term clinical scores, respectively).

### 2.4 EEG preprocessing

Three minutes of relatively artifact‐free EEG epochs were extracted from the following 19 derivations: Fp1, Fp2, F7, F3, Fz, F4, F8, T7, C3, Cz, C4, T8, P7, P3, Pz, P4, P8, O1, and O2. These epochs were accumulated from six 30 s EEG epochs (Tokariev et al., 2021), band‐pass filtered within [0.4–45] Hz, downsampled to 100 Hz, converted to an average montage, and finally split into five frequency bands of interest: low‐δ [0.4–1.5] Hz, high‐δ [1.5–4] Hz, θ [4–8] Hz, α [8–13] Hz, and β [13–22] Hz (stage 1 in Figure 1). The filtering process was achieved by means of cascaded zero‐phase low‐pass and high‐pass infinite impulse response filters. Moreover, cortical signals were estimated from the EEG using the symmetric boundary element method and the dynamic statistical parametric mapping for the forward and inverse modeling, respectively (Tokariev et al., 2018; Tokariev et al., 2021; Tokariev et al., 2022; Yrjölä et al., 2021). This process utilized a realistic three‐shell newborn head model that is comprised of manually segmented scalp, skull, and intracranial volumes (Tokariev et al., 2021), and the conductivities were set to 0.43 S/m for the scalp, 0.2 S/m for the skull, and 1.79 S/m for the other intracranial volume (Tokariev et al., 2018; Yrjölä et al., 2021). The initial cortical source space was clustered into 58 cortical parcels as previously described for newborn EEG analyses (Abbas et al., 2021; Mehrkanoon et al., 2014; Stam et al., 2007). In addition, the parcels could be also grouped into four brain regions (frontal, central, temporal, and occipital) as needed in the respective analysis steps.

### 2.5 Group‐level dynamic functional connectivity

To investigate the temporal dynamics in cortico‐cortical interactions, we first computed the individual, frequency‐specific connectivity matrices at a high temporal resolution, followed by their concatenation across the temporal dimension and over the cohort to form a group‐level tensor (stage 2 in Figure 1). First, the 3 min time‐courses of cortical signals were segmented by a 2 s sliding window with 50% overlap, resulting in 179 temporal segments for each cortical signal. The 2 second window duration was taken to capture at least one cycle of the lowest frequency (low‐δ oscillations), and to be short enough to reflect the rapid temporal dynamics in FCNs (Bassett & Sporns, 2017; Du et al., 2018; Haartsen et al., 2020; Panwar et al., 2021). A segment wise cross‐spectrum was computed between all pairs of cortical parcel signals using a discrete Fourier transform, which yielded estimates of phase–phase synchronization via the debiased weighted phase lag index (dwPLI) that is known for robustness against volume conduction effects (Yu, 2020). Then, a three‐way tensor of size 1653 × 179 × 5 was composed for each infant (or individual), corresponding to pairwise connectivity, time windows, and frequency bands, respectively (see Supplementary Section 1). Finally, group‐level dynamics were studied by concatenating these individual tensors across the temporal dimension to form a group‐level tensor of size 1653 × 179 *N* × 5 where *N* denotes the number of infants in that group.

The reliability of the connectivity matrices was improved by applying a fidelity mask. It is known that any limited number of recording EEG electrodes can yield reliable pairwise connectivity estimates from only a respective proportion of cortico‐cortical parcel pairs (Tokariev et al., 2021). Therefore, we used a simulation‐based procedure to exclude unreliable connections from further analysis (Tokariev et al., 2021). In brief, for each pair of cortical parcels, we generated perfectly synchronous signals (dwPLI = 1), whereas non‐synchronized activity was assigned to the rest of the parcels. After that, this synthetic cortical activity was reconstructed into EEG (forward modeling) and reconstructed back into cortical signals (inverse modeling). This procedure was repeated 500 times and the mean of reconstructed connectivity values for the initially synchronous pair of parcels was compared to surrogate connectivity from all other parcels and across all iterations. The reliability of a connection was determined by checking if its average connectivity was above the 99th percentile of surrogates. This led to a binary template (fidelity mask) that rejected unreliable connections (525 common to all subjects) from the group‐level tensor; hence, reducing its effective size to 1128 × 179 *N* × 5.

### 2.6 Latent network extraction

The global network structure within a group can be thought of as latent networks that are not directly observable from empirical measurements; hence, they require careful distillation. We extracted a latent representation from the group‐level tensor using non‐negative matrix factorization (NMF); a low‐rank matrix approximation tool that is appropriate for non‐negative data such as cortico‐cortical interactions (Mahyari et al., 2017; Zhou, Kang, Cong, Li, & X., 2020) (Stage 3 in Figure 1). The NMF technique was selected because it provides robust reconstruction results when compared to PCA and ICA (Thompson et al., 2020; Zhou, Kang, Cong, & Li, 2020), and because of its effectiveness in suppressing intersubject variability (Calesella et al., 2020; Calesella et al., 2021). We applied the NMF to the group tensor's frequency slices and the resultant estimates were aggregated back to their original tensor form (see Supplementary Section 2). Furthermore, the NMF rank, or model order, was automatically selected using an entropy‐based technique to avoid under and over‐fitting problems (see Supplementary Section 4.1). The selected NMF orders for the five frequency bands ([low‐δ, high‐δ, θ, α, β]) in the HC/AED group were: [19, 19, 20, 20, 20]/[19, 20, 21, 20, 21] and [18, 21, 20, 20, 23]/[20, 21, 19, 19, 21] for the infants with short‐ and long‐term neurobehavioral assessments, respectively.

### 2.7 Latent network decomposition

The extracted group‐level latent mdFCNs were decomposed into a set of components by canonical polyadic decomposition (CPD), which is a tensor‐generalization of the conventional matrix singular value decomposition (Cong et al., 2015; Zhu, Liu, Ye, et al., 2020) (stage 4 in Figure 1). The CPD defines the latent mdFCNs as a sum of tensors with each being the outer product of three non‐negative vectors describing pairwise connections, time/subject, and spectral factors (see Supplementary Section 3). In brief, it is mainly intended to break the latent mdFCNs into subnetworks (Chantal et al., 2021) to reveal patterns in the complex network feature space that could be linked to neurobehavioral phenotypes (Dron et al., 2021). Moreover, the CPD number of components was automatically selected using the introduced entropy‐based technique to suppress any remaining intersubject variability and noise (see Supplementary Section 4.2). The selected number of components was 14 and 13 for the infants with short‐ and long‐term clinical scores, respectively. Finally, to describe connectivity across subjects, the temporal/subject factors were block averaged; hence, the CPD components were changed to describe connectivity across pairwise connections, subjects, and frequency bands. Note that the FCNs correlations are computed for time‐invariant neurobehavioral phenotypes; therefore, this procedure removes direct measures of temporal dynamics per se, though they had been earlier used to accurately incorporate the signal's non‐stationarity (Al‐Sa'd et al., 2021; Al‐Sa'd & Boashash, 2019; Haartsen et al., 2020).

### 2.8 Component selection and network reconstruction

We selected a subset of the decomposed components for network reconstruction to analyze the FCNs with the strongest links to phenotypes (stage 5 in Figure 1). Since the frequency slices of the decomposed tensors are independent, we performed the component selection and network reconstruction procedures per frequency band. First, we formalized the component selection problem in a binary manner to exhaustively test every possible reconstruction configuration. Besides, we standardized the connectivity patterns of every subject by removing their median and setting their median absolute deviation to one. Afterward, for every reconstruction configuration, the relevance of the network was measured by correlating its standardized connectivity patterns across the infants with their clinical scores. Furthermore, the set of components corresponding to the best reconstruction strategy was found by maximizing the number of connections yielding significant correlations (network density), the averaged correlation of those significant connections, and the standardized connectivity goodness of fit to the clinical scores when regressed by linear lines. These criteria were combined into a fitness function via their geometric mean (see Figure 6a where the concise set of selected components reveals a global group structure that is associated with the infants' neurobehavioral phenotypes). We validated the reconstructed FCNs by a permutation test; nonetheless, to further ensure the mdFCN potency, we discarded densities below 2.5%. Finally, we repeated the component selection and network reconstruction procedures for every score and for both positively and negatively correlated networks.

### 2.9 Analysis of multiplex networks

A multiplex network is a system of interactions (edges) within a population (nodes) that is fixed at different attributes (layers) (Buldú & Porter, 2018; Kivelä et al., 2014; Vaiana & Muldoon, 2020). In this work, a multiplex brain network describes the FCNs among parcel regions at five frequency bands (Amoroso et al., 2019; Makarov et al., 2018; Rocca et al., 2020; Rubinov & Sporns, 2010). The multiplex network representation is convenient because it allows extracting node, layer, and edge structural features that can express the differences between the HC and AED groups in a meaningful concise manner (stage 6 in Figure 1). First, we averaged the reconstructed networks across the subjects' dimension and matricized their pairwise connections. This process requires folding the connectivity values back to their upper triangular position, inserting zeros for the omitted unreliable connections identified by the fidelity matrix, and adding a mirror image of the upper triangle of the matrix to its lower half. After that, we computed adjacency matrices for the five frequency bands and formed a multiplex unweighted network (see Supplementary Section 5.1). Furthermore, we calculated the following node, layer, and edge multiplexity structural measures (Battiston et al., 2014; Battiston et al., 2017; Rubinov & Sporns, 2010): normalized overlapping degree, multiplex participation coefficient, pairwise multiplexity, hamming distance, total overlap ratio, and an overall edge intersection index (see Supplementary Sections 5.2–5.4). Finally, we averaged the measures across all the infants' scores and for both positively and negatively correlated FCNs to compare the HC cartography to the AED group.

### 2.10 Statistics and reproducibility

The mdFCN relevance with phenotypes was measured by correlation using Spearman's rank coefficient with the infants' conceptional age acting as a covariate. We corrected for multiple comparisons using the Benjamini–Hochberg procedure at 5% significance. Moreover, we generated a null distribution for both correlation directions by permuting the infants' pairwise connections 10,000 times, calculating the number of connections yielding significant correlations (connection density) at every permutation, and by computing the densities' frequency. We calculated the probability of finding statistically significant connections by chance and compared those against the reported findings. Finally, group differences in the clinical scores were measured by a two‐tailed Wilcoxon rank sum test at 5% significance.

## 3 RESULTS

### 3.1 Analysis of mdFCNs

**FIGURE 2:**
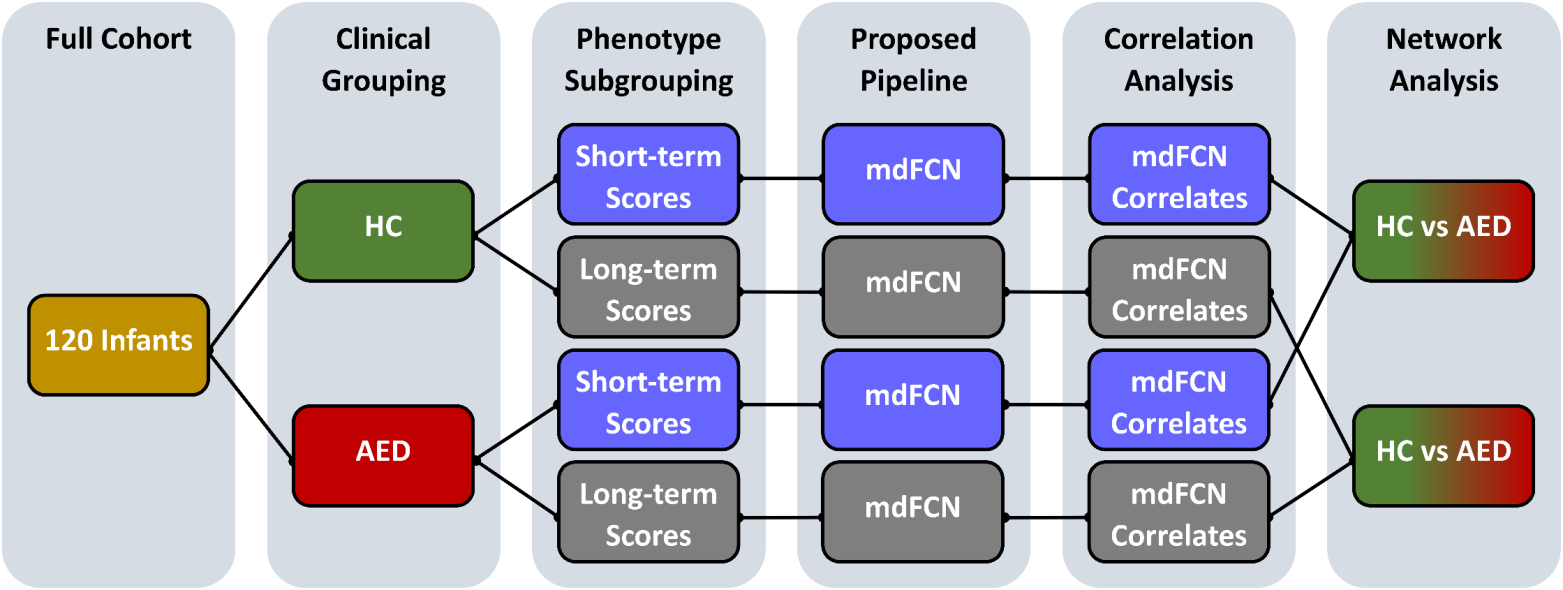
The study comparative analysis hierarchy. The comparative analysis between the revealed brain networks of the healthy control (HC) and the infants exposed *in utero* to maternal treatment with antiepileptic drugs (AED) includes correlations to neurobehavioral scores and network structural comparisons. The multiplex dynamic functional connectivity network (mdFCN) stages 2–4 in Figure 1 were computed four times (four phenotype subgroups) and stages 5–6 were computed 14 times (two short‐term and five long‐term neurobehavioral scores for two clinical groups).

We analyzed mdFCNs from two groups of infants: healthy controls (HC, *N* = 68) and infants exposed *in utero* to maternal treatment with antiepileptic drugs (AED, *N* = 52) (Tokariev et al., 2021; Videman et al., 2016) with gestational age (average ± standard deviation) of 40.3 ± 1.1 and 40.1 ± 1.4 weeks, respectively. The EEG signals were captured near term age (HC group: 42.2 ± 0.9 weeks; AED group: 42.1 ± 0.9 weeks) for about an hour that included both cardinal sleep states, AS and QS, and we extracted 3 min of relatively artifact‐free epochs (per subject and sleep state). We preprocessed the extracted epochs by band‐pass filtering and downsampling, split their spectra into five frequency bands of interest: low‐delta (0.4–1.5 Hz), high‐delta (1.5–4 Hz), theta (4–8 Hz), alpha (8–13 Hz), and beta (13–22 Hz), and computed frequency‐specific source space signals using a realistic infant head model (stage 1 in Figure 1). We also generated cortico‐cortical mdFCNs at high temporal resolution to be concatenated into one large cohort‐level tensor (stage 2 in Figure 1), extracted a latent representation from this cohort‐level tensor by matrix factorization (Stage 3 in Figure 1), decomposed it into a set of components via tensor rank decomposition (stage 4 in Figure 1), and selected a concise subset of these components for reconstruction (stage 5 in Figure 1). The reconstructed mdFCNs were then correlated to several neurobehavioral assessments conducted during the neonatal period (short‐term) and at 2 years of age (long‐term), as well as compared between the two clinical groups (HC vs. AED, see Figure 2 for the study analysis hierarchy).

### 3.2 The mdFCN correlates with neurobehavioral phenotypes

**FIGURE 3:**
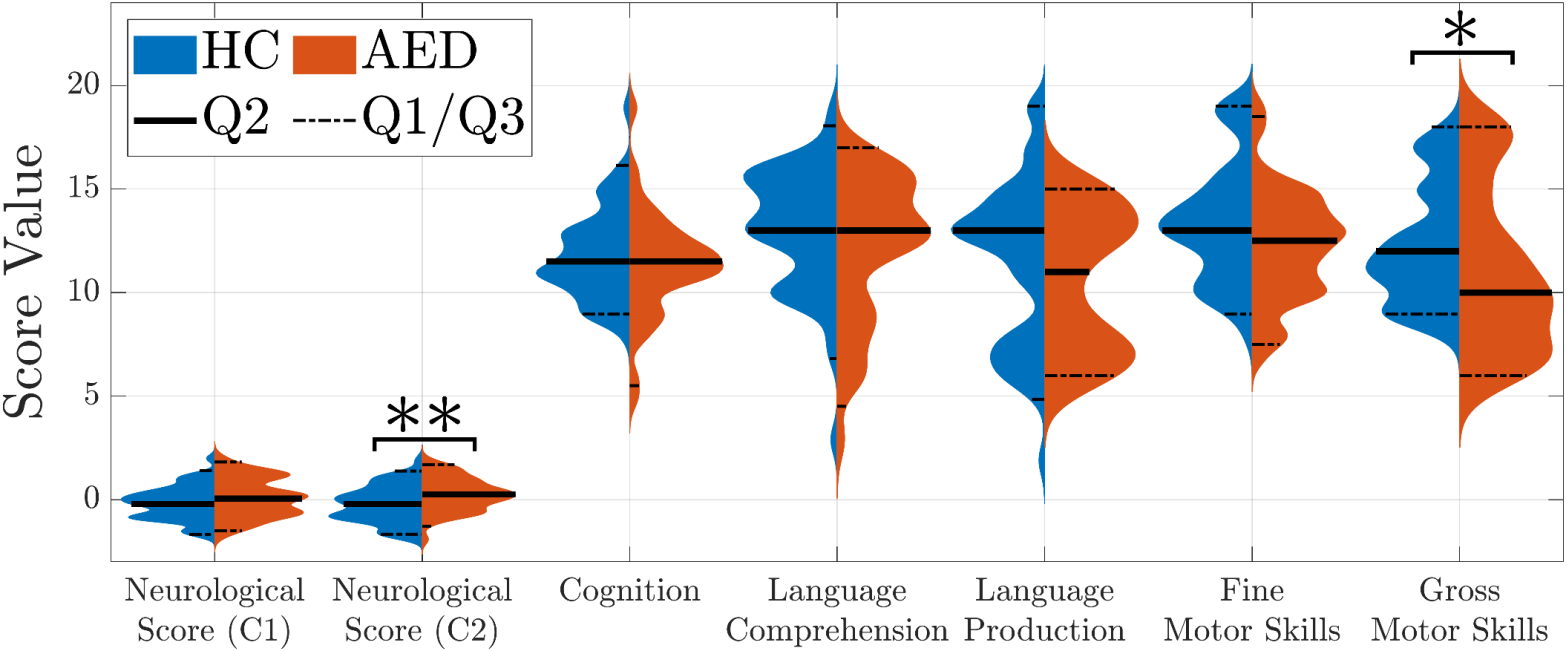
Neurobehavioral scores of the healthy controls (HC) and the infants exposed *in utero* to maternal treatment with antiepileptic drugs (AED). Group differences in the clinical scores were measured by a Wilcoxon rank sum test at 5% significance. Statistically significant differences with *p*‐values below 0.05 and 0.01 are indicated by * and **, respectively. The scores' distributions are smoothed using normal kernels and plotted along with their 25% (Q1), 50% (median or Q2), and 75% (Q3) percentiles.

The reconstructed mdFCNs in each clinical group (HC and AED) were correlated to two short‐term neurological scores (Neurological C1 and C2 which correlate with later motor and cognitive/social performances, respectively (Tokariev et al., 2018; Tokariev et al., 2021; Yrjölä et al., 2021)) and five long‐term clinical scores (cognition, language comprehension, language production, fine motor skills, and gross motor skills). The variations in clinical scores among the groups show statistical insignificance across most outcomes, barring the neurological C2 and gross motor skills (see Figure 3 for the scores' distributions and their group differences). Therefore, any dissimilarity between the groups, identified by their mdFCNs, is mostly indicative of distinctions in their functional network configurations and it is not a consequence of differences in their clinical scores.

**FIGURE 4:**
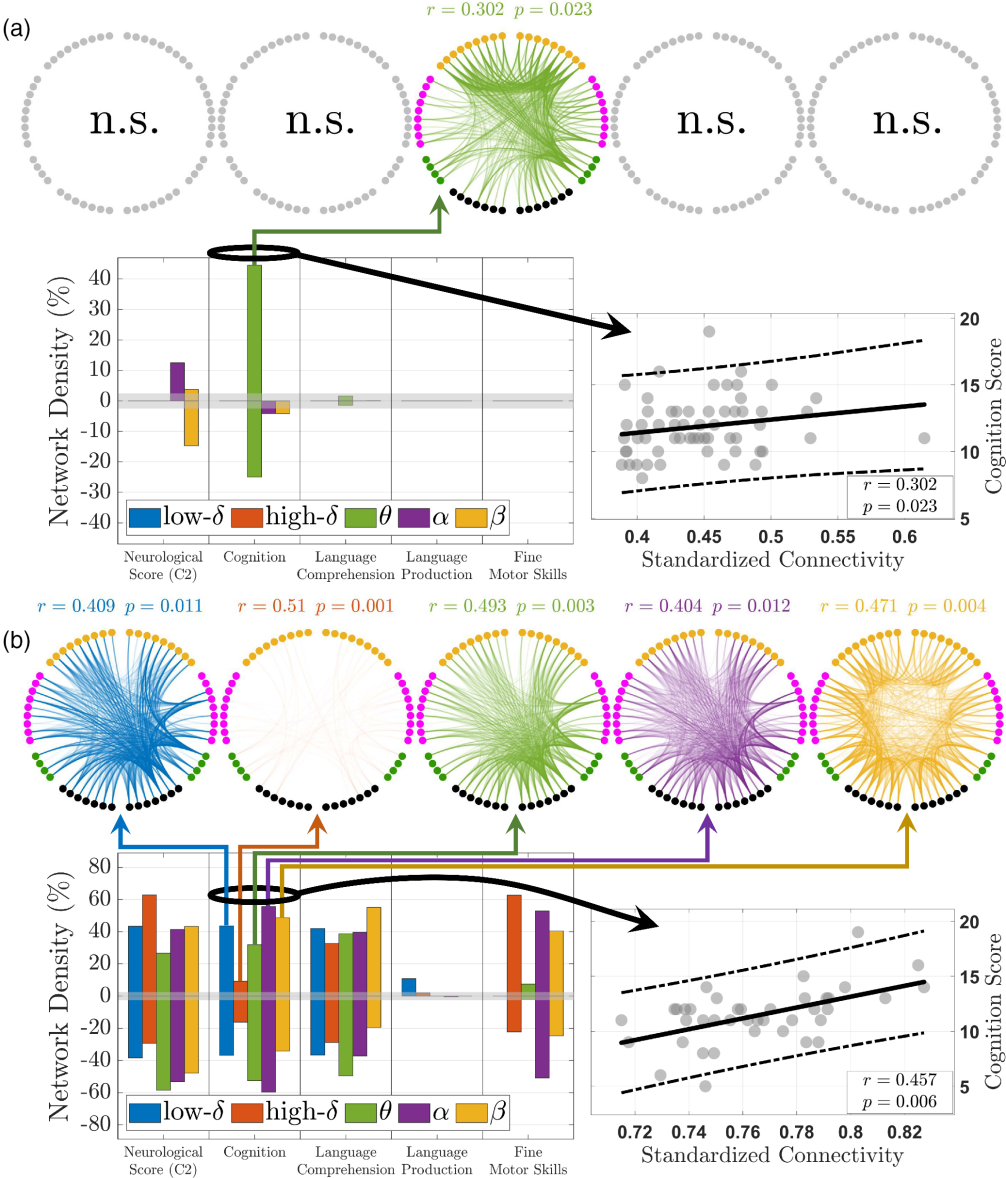
The multiplex dynamic functional connectivity network (mdFCN) correlation analysis. (a) The mdFCN correlates with the neurobehavioral phenotypes in the healthy controls (HC group). (b) The mdFCN correlates with the neurobehavioral phenotypes in the infants exposed *in utero* to maternal treatment with antiepileptic drugs (AED group). The network densities are illustrated for five clinical scores, for every frequency band of interest, with a focus on the mdFCN that is positively correlated with the long‐term cognition outcome (see Supplementary Figures 4–8,11, and 12 for the complete set of results). Positive/negative densities denote the relative number of connections yielding significant positive/negative correlations with the clinical scores. The brain connections' color transparency is enhanced to ease visualization by squaring the standardized connectivity values and their frequency combined linear regression outputs are shown as solid lines along with their 95% confidence intervals in dashed lines. Densities below the 2.5% mark, shown in gray, are considered nonsignificant (n.s.) and are discarded from the analysis.

We measured the mdFCNs correlation by the Spearman's rank coefficient with the infants' conceptional age as a covariate and corrected the multiple comparisons by the Benjamini–Hochberg procedure at 5% significance. The correlation analysis shows statistically significant brain connections existing in both the HC and AED groups, but with varying correlation levels and network densities (i.e., the ratio of connections yielding significant correlations) depending on the clinical scores and frequency bands (see Figure 4 for focus on the mdFCN that was positively correlated with the cognition outcome and Supplementary Figures 4–8, 11, and 12 for the complete set of results). For instance, the results in Figure 4a suggest that the HC networks are linked to only few neurobehavioral scores with densities reaching up to 14.7% and 44.5% for the neurological C2 and cognition scores, respectively. In contrast, the AED networks in Figure 4b show a more consistent and higher number of significant connections when correlated with most neurobehavioral outcomes. Specifically, they demonstrate network densities reaching up to 62.8% with the language production score being the only exception at 10.8%. Moreover, by comparing the HC to the AED results, one notes sound differences in the networks' frequency behavior. On the one hand, the mdFCN correlates in the HC group are frequency selective because statistically significant results were confined to few frequency bands. On the other hand, the AED networks appear not to favor any operating frequency except when correlated with the language production score where the low‐delta band was the sole contributor. Finally, the AED reconstructed mdFCNs exhibit higher spectral similarity (pairwise connections that are similar at different frequency bands) and higher correlation averages when compared to the HC group (see Supplementary Figures 11 and 12, HC: *r* = 0.399 at *p* = 0.007 and *r* = −0.411 at *p* = 0.005; AED: *r* = 0.428 at *p* = 0.009 and *r* = −0.422 at *p* = 0.009). Altogether, these observations suggest that brain networks in healthy infants are less associated with their neurobehavioral phenotypes, in comparison to the ones exposed *in utero* to antiepileptic medications, perhaps, due to their intrinsic high intersubject variability and spectral diversity.

### 3.3 The mdFCN structure reveals group‐level differences

**FIGURE 5:**
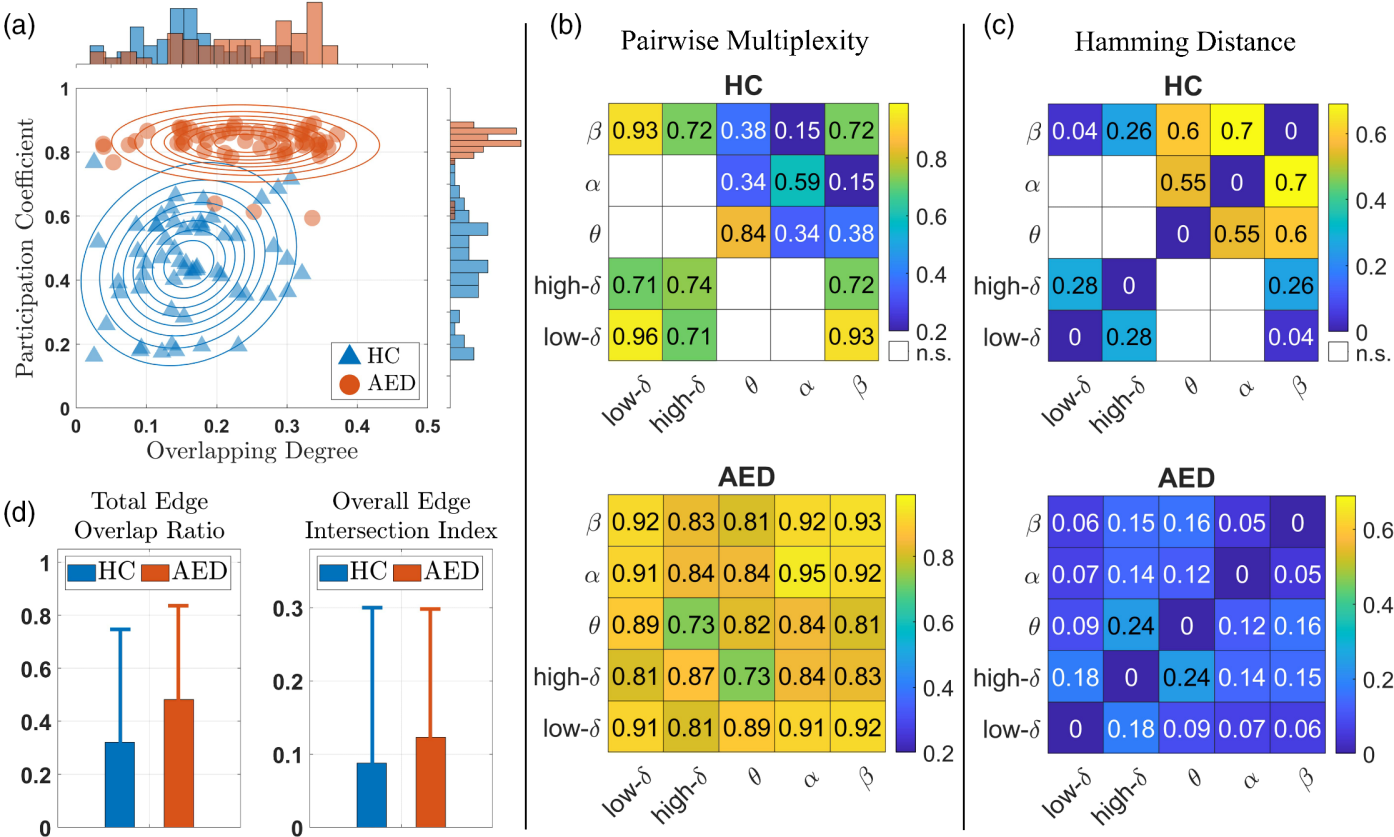
Comparing the multiplex structure of the multiplex dynamic functional connectivity networks (mdFCNs) in the healthy control group (HC) to the infants exposed *in utero* to maternal treatment with antiepileptic drugs (AED). (a) The distribution of node or spatial features (participation coefficient and overlapping degree). (b) The network layer or spectral similarity (pairwise multiplexity). (c) The mdFCN spectral similarity (hamming distance). (d) The mdFCN edge or pairwise connection features (total edge overlap ratio and overall edge intersection index).

We next asked if there were any major structural differences between the revealed HC and AED brain networks. To answer this question, we quantified the multiplex structure of the significantly correlated mdFCNs using various measures such as the normalized overlapping degree, multiplex participation coefficient, pairwise multiplexity, hamming distance, total overlap ratio, and the overall edge intersection index (Rubinov & Sporns, 2010) (stage 6 in Figure 1). The computed measures were then averaged across all the neurobehavioral outcomes and for both positively and negatively correlated mdFCNs to compare the HC network cartography to the AED group (see Figure 5). First, by examining the results in Figure 5a, there is a clear distinction between the two groups in the distributions of their multiplex structure. On the one hand, the participation coefficient distribution in the HC infants is centered at 0.45 ± 0.15 (average ± standard deviation), which is suggestive of a mixed network (Battiston et al., 2014; Rubinov & Sporns, 2010), that is, neither multiplex nor focused (see Supplementary Section 5.2). Besides, the distribution's high deviation implies the existence of cortical regions with varying levels of participation, that is, the regions' pairwise connections operate neither uniformly nor strictly within a few frequency bands. On the other hand, the coefficient's distribution in the AED infants is centered around 0.83 ± 0.06 suggesting a strictly multiplex network with less variability in nodal participation, that is, the regions' pairwise connections coexist at different frequency bands. Moreover, the overlapping degree distributions indicate the emergence of network hubs that are clearly present in the AED group only (AED: 0.24 average with −0.54 skewness, HC: 0.16 average with 0.35 skewness). Another discrimination in the groups' neural organization is demonstrated by the similarity in their layer or spectral content (Figure 5b,c). The results demonstrate that brain networks in the AED group have much higher intra‐layer similarity when compared to the HCs. Specifically, they yield an averaged pairwise multiplexity of 0.86 ± 0.06 and averaged Hamming distance of 0.1 ± 0.07 when compared to the HC levels at 0.6 ± 0.27 and 0.29 ± 0.28, respectively. Finally, alikeness in the HC and AED network edges (Figure 5d) conveys that the probability of finding a statistically significant pairwise connection that coexists in all frequency bands is higher in the AED multiplex. Additionally, the results show that mdFCN correlation to neurobehavioral phenotypes is higher in the AED group, which was also confirmed by the correlation analysis (Figure 4 and Supplementary Figures 11 and 12).

### 3.4 Effectiveness of the proposed mdFCN analysis pipeline

**FIGURE 6:**
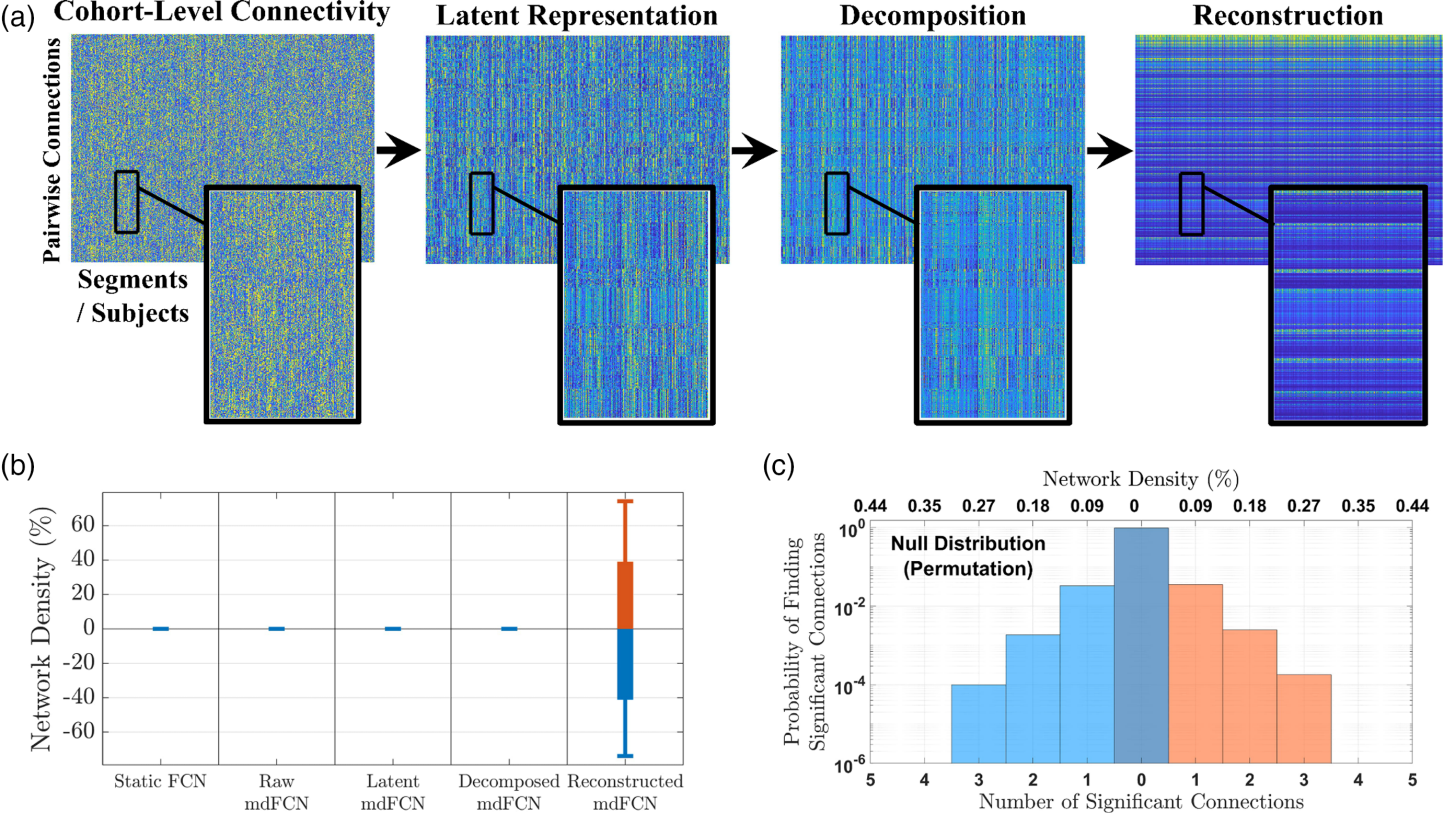
Effectiveness of the proposed analysis pipeline in linking multiplex dynamic functional connectivity networks (mdFCNs) to phenotypes. (a) The group‐level processing from raw connectivity (left) to network reconstruction (right). Note how the high temporal and interindividual variability (analytical noise) in the cohort‐level connectivity matrix (low‐delta frequency, antiepileptic drug [AED] group) has rapidly decreased during the process, leading to the last stage where visually clear patterns emerge corresponding to a global network structure seen at the group level (see Supplementary Figure 13 for the network reconstruction strategy). (b) The pipeline verification using the long‐term cognition score (see Supplementary Figure 8b, 9, and 10 for the complete set of results). Positive/negative densities denote the relative number of connections yielding significant positive/negative correlations with the clinical score. (c) The network density null distribution for both correlation directions. The null distribution was generated by permuting the infants' pairwise connections 10,000 times, calculating the number of connections yielding significant correlations at every permutation, and computing the densities' frequency. Note that the y‐axis in (c) is shown on a logarithmic scale to ease visualization and interpretation.

The proposed pipeline relies on the extraction, decomposition, and reconstruction of latent features for linking the infants' mdFCNs to phenotypes. This group‐level processing from raw connectivity (Figure 6a, left) to network reconstruction (Figure 6a, right) was developed to reduce the high temporal and interindividual variability, which is often considered as noise in the network analysis; thereby the group‐level analysis was assumed to reveal the global group structure that associates with the infants' neurobehavioral phenotypes. Nonetheless, to explicitly verify the pipeline's effectiveness, we correlated its intermediate outputs with the infants' neurobehavioral outcomes. Then, we computed the resulting number of connections yielding significant correlations, returning network densities averaged across the five frequency bands of interest, and we compared those to the standard static FCN (see Figure 6b for focus on verifying the mdFCN correlates with the cognition outcome and Supplementary Figures 8b, 9, and 10 for the complete set of results). The verification results confirm that statistically significant correlations are only found using the reconstructed networks. In other words, the lack of statistically significant results when using the pipeline's intermediate outputs or the static FCN showcases that each stage in the mdFCN framework is essential to achieve local goals that, when combined, allow for uncovering latent network properties that correlate with the clinical scores. Moreover, the network density null distribution, generated by permutation testing (Figure 6c), validates the study findings and the proposed mdFCN analysis pipeline. Specifically, the validation results show that the probability of finding statistically significant connections by chance decays exponentially with the number of connections, with a 0.018% chance of finding three statistically significant connections by mistake. Therefore, network densities above 0.27% are very likely to be meaningful. Nevertheless, to ensure precision we imposed a stricter criterion when analyzing the mdFCN correlates by only considering networks with at least 2.5% correlation density (see the minimum network density mark shown in gray in Figure 4).

## 4 DISCUSSION

The present study shows robust network constellations in the rapid dynamics of newborn cortical activity that strongly correlate with several neurobehavioral phenotypes. The novel pipeline presents an end‐to‐end solution for extracting mdFCNs at the group level, to be applied for phenotypic correlations at the individual level. The findings are consistent with prior studies on adult subjects showing that accounting for temporal dynamics and coincident network configurations at multiple frequencies allows extracting informative structures that correlate with phenotypes (Carotenuto et al., 2022; de la Cruz et al., 2021; García et al., 2013; López‐Vicente et al., 2021; Shi et al., 2018; Tessadori et al., 2021; Tewarie et al., 2019; Vaidya & Gordon, 2013; Zhu, Liu, Ye, et al., 2020).

Our work extends prior knowledge by showing that intrinsic multiplex network characteristics are indeed latent properties in the high‐level noise, and they can be uncovered by the developed framework in a robust manner. Specifically, prior studies in infants have only examined temporally and/or spectrally static networks (Omidvarnia et al., 2015; Tokariev et al., 2019; Westende et al., 2020; Yrjölä et al., 2022), whereas some studies in adults have explored the combination of spectrally distributed networks (Buldú & Porter, 2018; Vaiana & Muldoon, 2020; Zhu, Liu, Ye, et al., 2020) and their dynamic changes (Chantal et al., 2021; Esfahlani et al., 2020; Mahyari et al., 2017; Mehrkanoon et al., 2014; Tewarie et al., 2019; Zhu, Liu, Mathiak, et al., 2020; Zhu, Liu, Ye, et al., 2020). In contrast, the proposed mdFCN analysis pipeline fully exploits the network dynamic multiplexity and introduces significant improvements. First, it extracts latent representations from group‐level mdFCNs to effectively suppress noise and/or intersubject variations. This is essential to uncover the group‐level structure (Mehrkanoon et al., 2021; Zhu, Liu, Mathiak, et al., 2020), to support the network decomposition task because high variability leads to superfluous components, and to yield reliable/repeatable findings (Panwar et al., 2021; Shellhaas et al., 2022; Westende et al., 2020). The latter is paramount because phase‐based functional connectivity measures are challenged with relatively lower test–retest reliability when compared to amplitude‐based connectivity metrics (Colclough et al., 2016). Nonetheless, here we use phase–phase synchronization because previous studies showed its links to neurodevelopment in infants (Tokariev et al., 2021; Yrjölä et al., 2021) and higher cognitive functions in adults (Hirvonen et al., 2018; Lobier et al., 2018; Williams et al., 2023). Second, the mdFCN analysis pipeline guides the extraction/decomposition of networks by an automatic model‐order selection procedure to avoid undescriptive group‐level features (under‐fitting) or the inclusion of overt intersubject differences and noise (over‐fitting). Third, the proposed framework automatically finds a subset of the decomposed networks for reconstruction, which allows identifying FCNs with much stronger and wider relationships to neurobehavioral phenotypes than before (Tokariev et al., 2018; Tokariev et al., 2019; Tokariev et al., 2021; Tokariev et al., 2022; Videman et al., 2016; Yrjölä et al., 2021; Yrjölä et al., 2022), and demonstrates the wide‐scale effects of *in utero* drug exposure (Tokariev et al., 2021; Tokariev et al., 2022; Videman et al., 2016). Finally, it adopts multiplex network representations (Brookes et al., 2016; Buldú & Porter, 2018) and various graph metrics (De Domenico et al., 2016; Yu et al., 2017) to express structural multiplexity differences in the groups' mdFCNs (Mandke et al., 2018) at the edge, nodal, and layer levels, in a meaningful concise manner (Battiston et al., 2014; Battiston et al., 2017; Rubinov & Sporns, 2010). Altogether, the novel mdFCN pipeline provides an end‐to‐end solution for an objective estimation of neurobehaviorally meaningful network constellations.

The spatial architectures of the underlying structural networks supporting the core FCNs organize during late pregnancy and early postnatal life in an activity‐dependent manner (Oldham & Fornito, 2019). An age‐appropriate activity is essential for supporting the organization of early FCNs that will develop further to sustain lifelong neurocognitive performance (Oldham & Fornito, 2019). The sensitivity of early neuronal activity to homeostatic and environmental effects implies that early FCNs are exquisitely sensitive to a range of early‐life medical adversities, such as *in utero* exposures to maternal illness or drug treatments (Tokariev et al., 2021; Tokariev et al., 2022; Videman et al., 2016), prematurity (Omidvarnia et al., 2015; Tokariev et al., 2018; Tokariev et al., 2019; Uchitel et al., 2022; Westende et al., 2020; Yrjölä et al., 2021; Yrjölä et al., 2022), or perinatal events (Abbas et al., 2021; Rocca et al., 2020). Understanding neurobehavioral and clinical correlates of the cortical activity networks in newborn infants, directly builds the systems‐level mechanistic understanding of how early neural activity supports the development of neurocognitive functions (Tokariev et al., 2021; Yrjölä et al., 2021). This also may directly support early diagnostics and interventions for better health outcomes (Oldham & Fornito, 2019; Tokariev et al., 2021; Yrjölä et al., 2021). The analysis methods developed thus far have been only able to show occult correlations between FCNs and clinical outcomes (Uchitel et al., 2022). Nevertheless, the developed mdFCN analysis pipeline overcomes this challenge by disclosing latent characteristics of infant cortical networks that support clinically important neurobehavioral success. Moreover, it reveals robust differences between cortical networks in healthy controls (HCs) versus infants who were exposed *in utero* to maternal treatment with antiepileptic drugs (AEDs). In brief, the mdFCN correlates indicated that statistically significant brain connections do exist in both the HC and AED infant groups, but with varying degrees depending on the neurobehavioral outcome and oscillatory frequency. Besides, a comparison of the infant groups showed that network‐phenotype correlations are much more pervasive in the AED infants compared to the HCs. For instance, neurobehavioral performance in the AED infants was related to more cortico‐cortical connections and more frequencies. Furthermore, the brain networks in both groups showed intra‐similar pairwise connections across the different operating frequencies. However, this trend was more prominent in the AED group suggesting a harmonious spectral behavior due to the *in utero* exposure to maternal drugs and greater variance in connectivity patterns among healthy infants. This might feel somewhat counterintuitive that wider correlations were found in infants after medical adversities; however, this observation is in line with prior results using static network analyses (Tokariev et al., 2021; Tokariev et al., 2022; Videman et al., 2016; Yrjölä et al., 2021). They are altogether compatible with the idea that mdFCNs hold constellations shared at the group level, and accordingly, higher flexibility in the uncompromised infants may allow more varying ways to carry out the same performance. Nevertheless, findings from both groups converge to the notion that the mdFCN pipeline is robustly extracting complex networks with neurobehavioral importance, and therefore, it may have significant utility as a systems‐level network biomarker in benchmarking developmental and therapeutic studies.

The present study has some limitations. First, resolving group‐level FCNs is unavoidably affected by the number of subjects that carry along intersubject variations. A greater number of subjects and/or data could yield more robust and generalizable estimates for the group's global structure or state; the effectiveness of the mdFCN pipeline cannot be guaranteed with scarce data. Second, component selection and network reconstruction (stage 5 in the mdFCN pipeline) may raise computational challenges because of its exhaustive search process.

The potential utility of the mdFCN pipeline is not limited to newborn EEG, but it can be applied to analyze recordings from older subjects, with minimal changes, as well as other data modalities, such as MEG (Gaudet et al., 2020; Sakkalis, 2011). Moreover, its fully data‐driven approach may prove to be valuable in extracting network constellations in a large range of contexts: In the fundamental neuroscience, the mdFCN pipeline may identify network behavior in the dynamically evolving abnormalities such as seizures or uncover the complex network interactions underlying behavioral or cognitive tasks. On the clinical side, the mdFCN analyses may support neuroscientifically reasoned diagnostic categories as a complement to the current phenomenological classifications in neuropsychiatric conditions, and finally, they might be useful as functional biomarkers in patient stratification or outcome measures in clinical trials.

## CONFLICT OF INTEREST STATEMENT

The authors declare no conflict of interests.

## Supporting information


**DATA S1.** Figures.

**DATA S2.** Supporting Information.

## Data Availability

The authors declare that the data supporting the findings of this study are available within the paper and its supplementary information files. The MATLAB scripts and functions developed for this work will be available on the corresponding author's GitHub page at https://github.com/Al-Sad.
