# Supplementary material for: Multiplex dynamic networks in the newborn brain disclose latent links with neurobehavioral phenotypes
Source: Hum Brain Mapp. 2024 Feb 5;45(2):e26610. doi: 10.1002/hbm.26610 (PMC10839739; doi:10.1002/hbm.26610)
Supplement: Supplementary file 1 — DATA S1. Figures. [file HBM-45-e26610-s001.pdf]

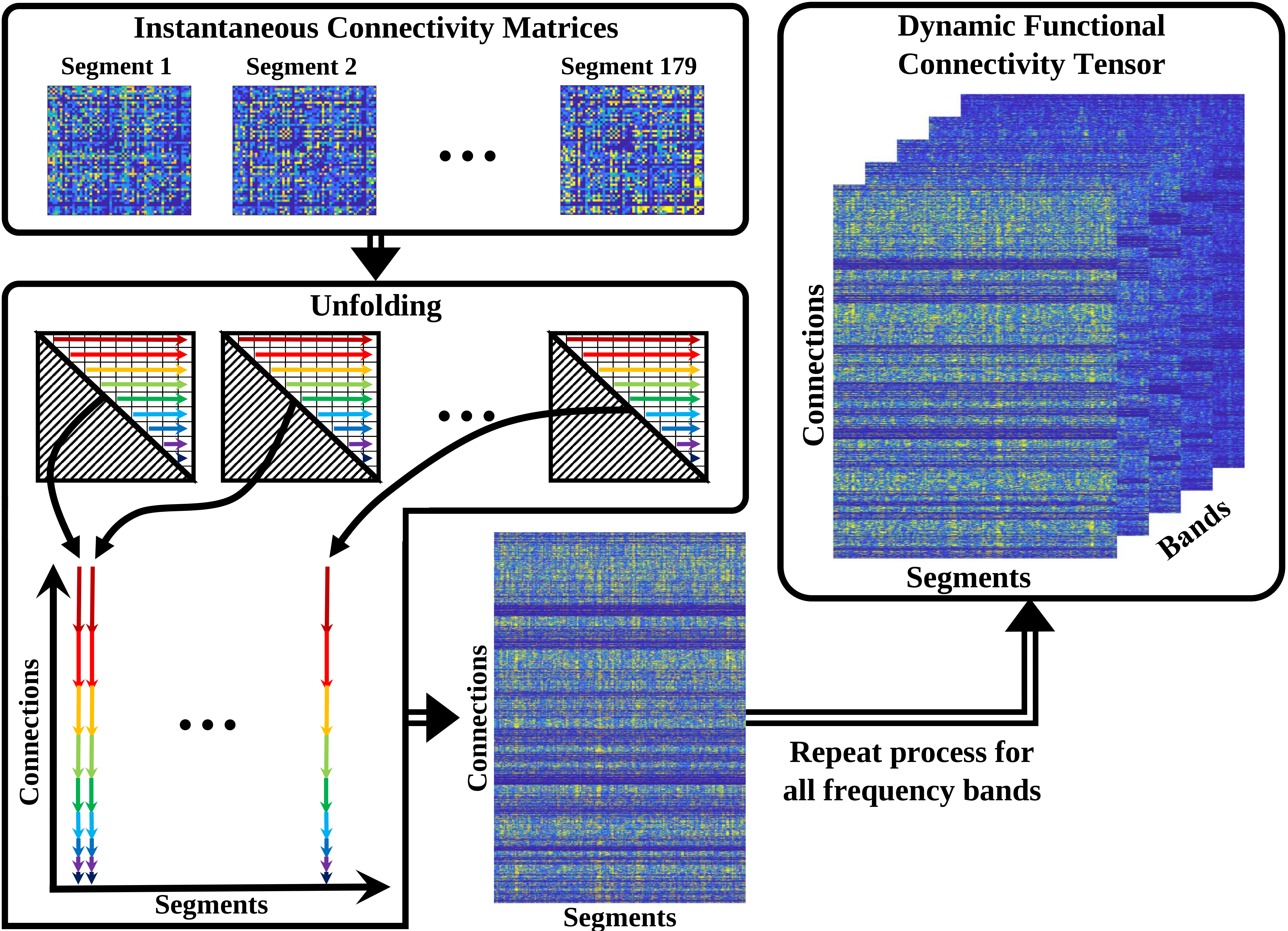

Instantaneous Connectivity Matrices
Segment 1
Segment 2
Segment 179
Unfolding
Connections
Segments
Connections
Segments
Repeat process for
all frequency bands
Dynamic Functional
Connectivity Tensor
Connections
Segments
Bands

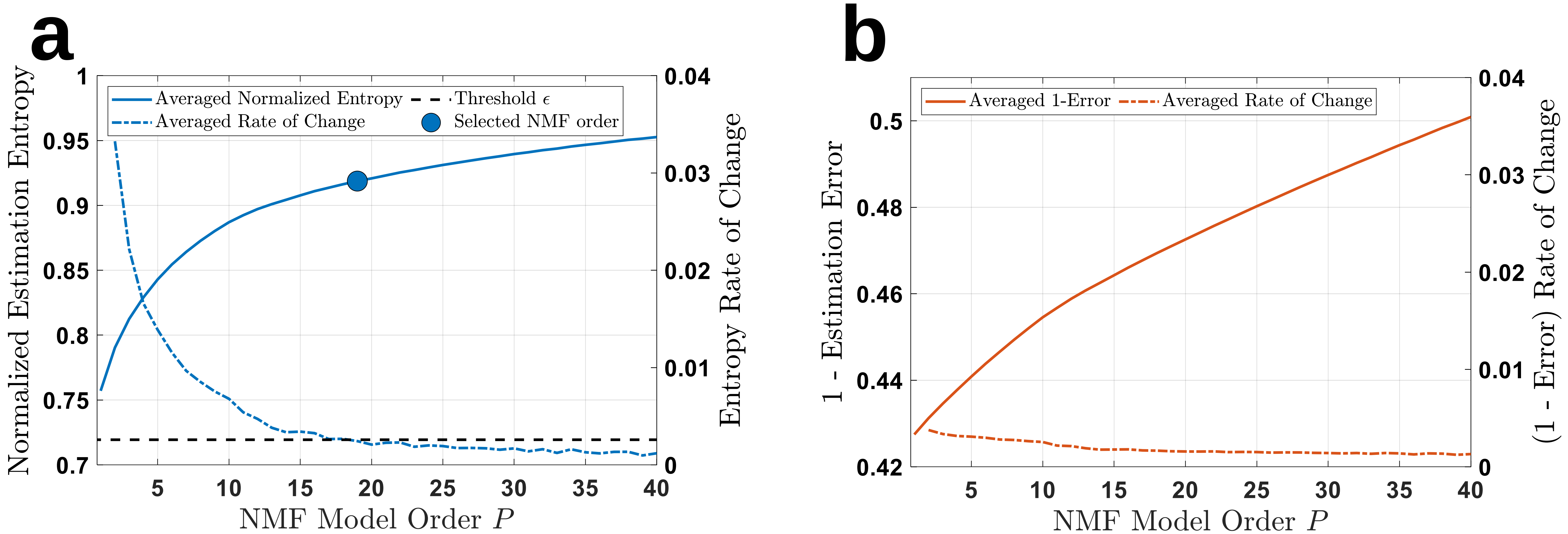
a
b
Averaged Normalized Entropy
Threshold ϵ
Averaged Rate of Change
Selected NMF order
Normalized Estimation Entropy
Entropy Rate of Change
NMF Model Order P
Averaged 1-Error
Averaged Rate of Change
1 - Estimation Error
(1 - Error) Rate of Change
NMF Model Order P

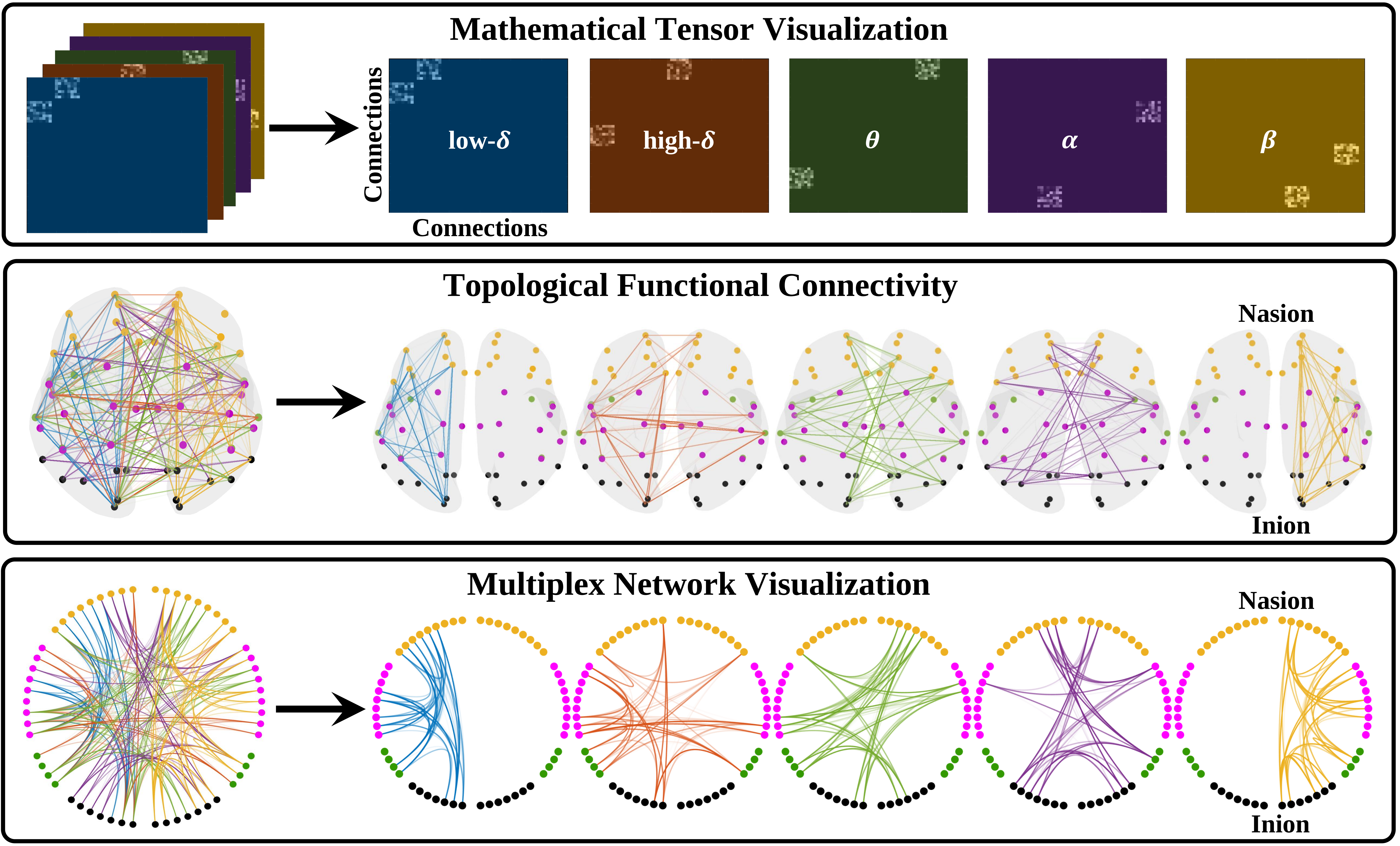

Mathematical Tensor Visualization
Connections
Connections
low-δ
high-δ
θ
α
β
Topological Functional Connectivity
Nasion
Inion
Multiplex Network Visualization
Nasion
Inion

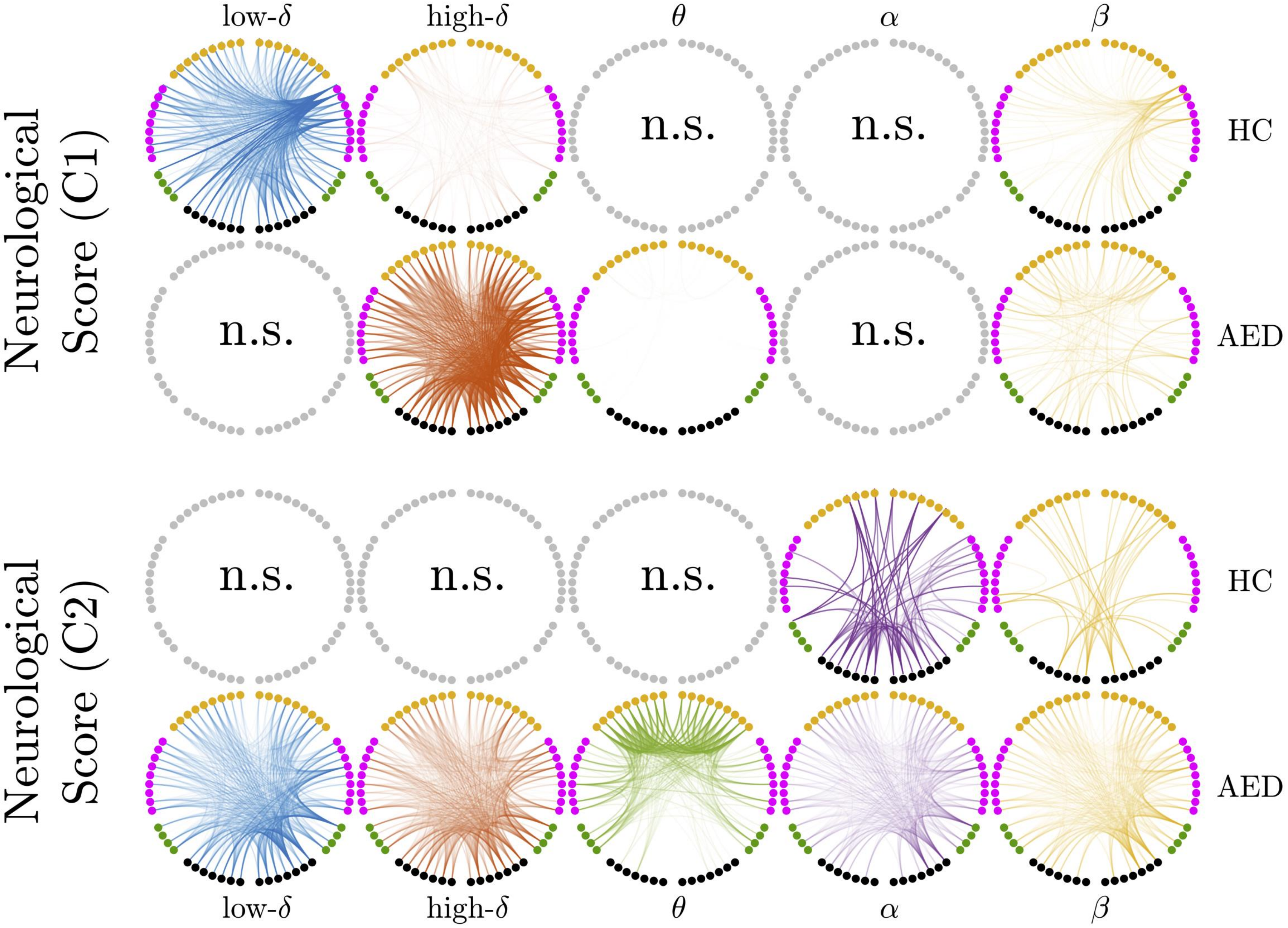

low-δ
high-δ
θ
α
β
Neurological Score (C1)
HC
AED
n.s.
n.s.
n.s.
n.s.
Neurological Score (C2)
HC
AED
n.s.
n.s.
n.s.
low-δ
high-δ
θ
α
β

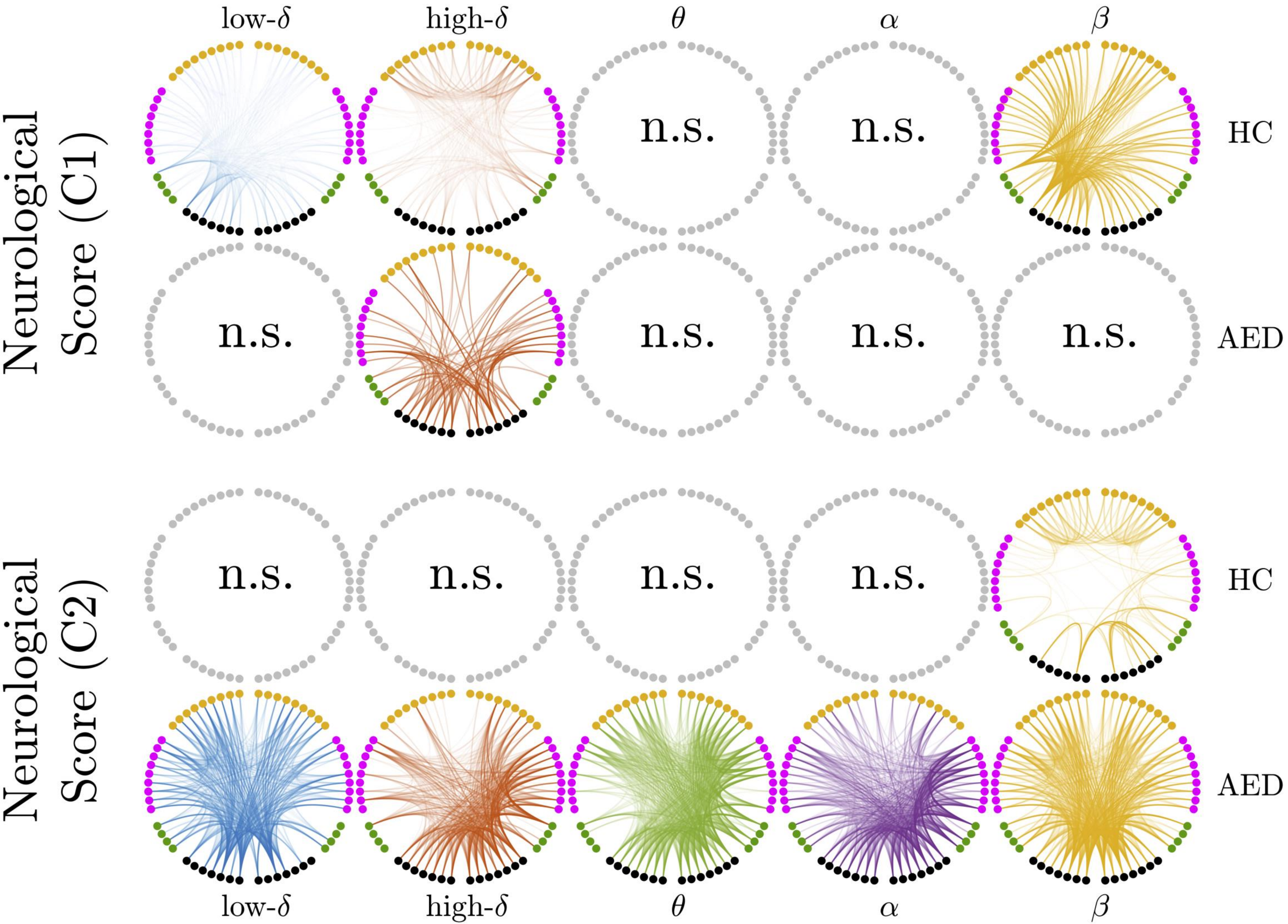

low-δ
high-δ
θ
α
β
Neurological Score (C1)
HC
AED
n.s.
n.s.
n.s.
n.s.
n.s.
n.s.
Neurological Score (C2)
HC
AED
n.s.
n.s.
n.s.
n.s.
low-δ
high-δ
θ
α
β

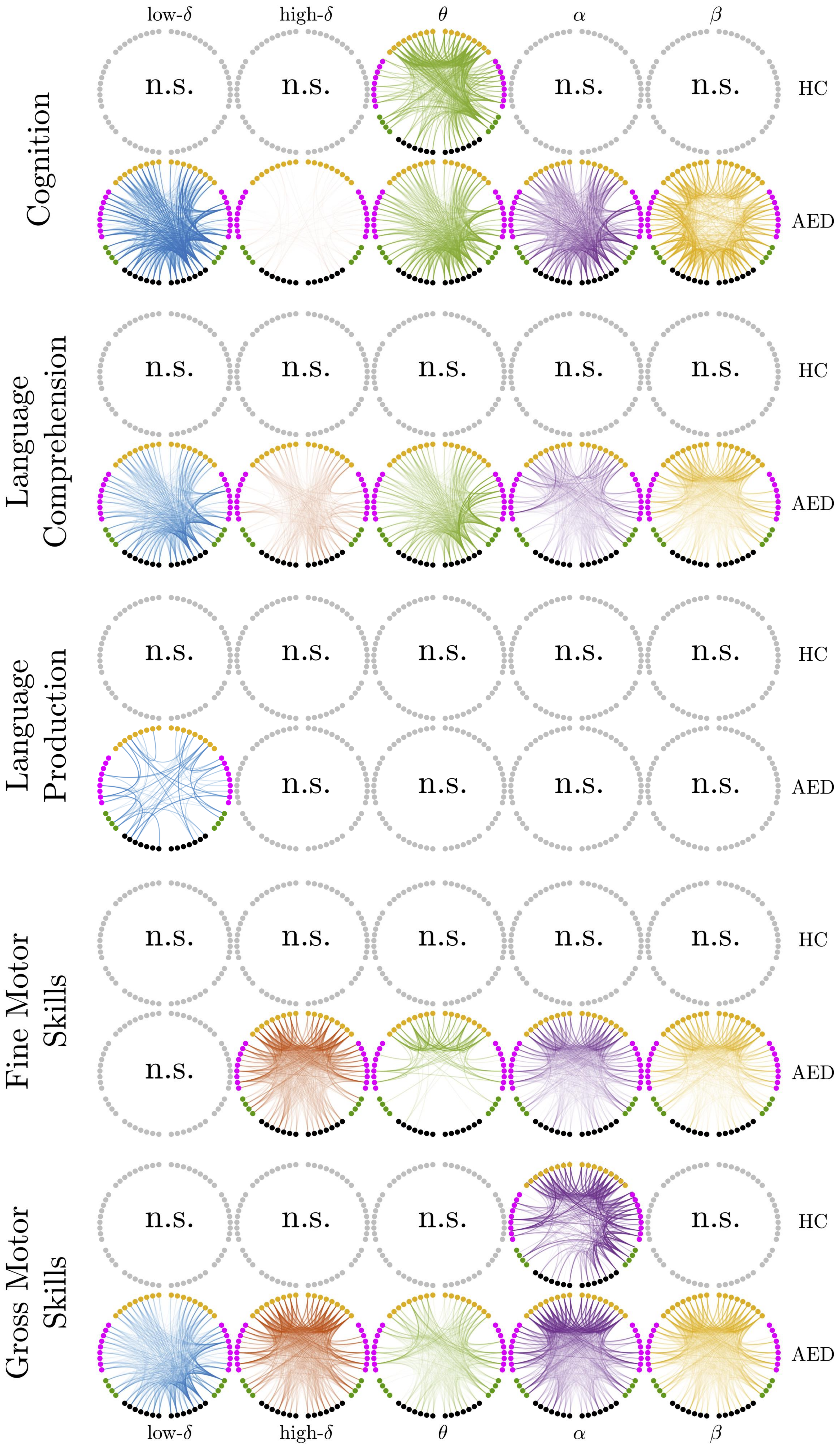
low-δ
high-δ
θ
α
β
Cognition
Language Comprehension
Language Production
Fine Motor Skills
Gross Motor Skills
HC
AED
n.s.

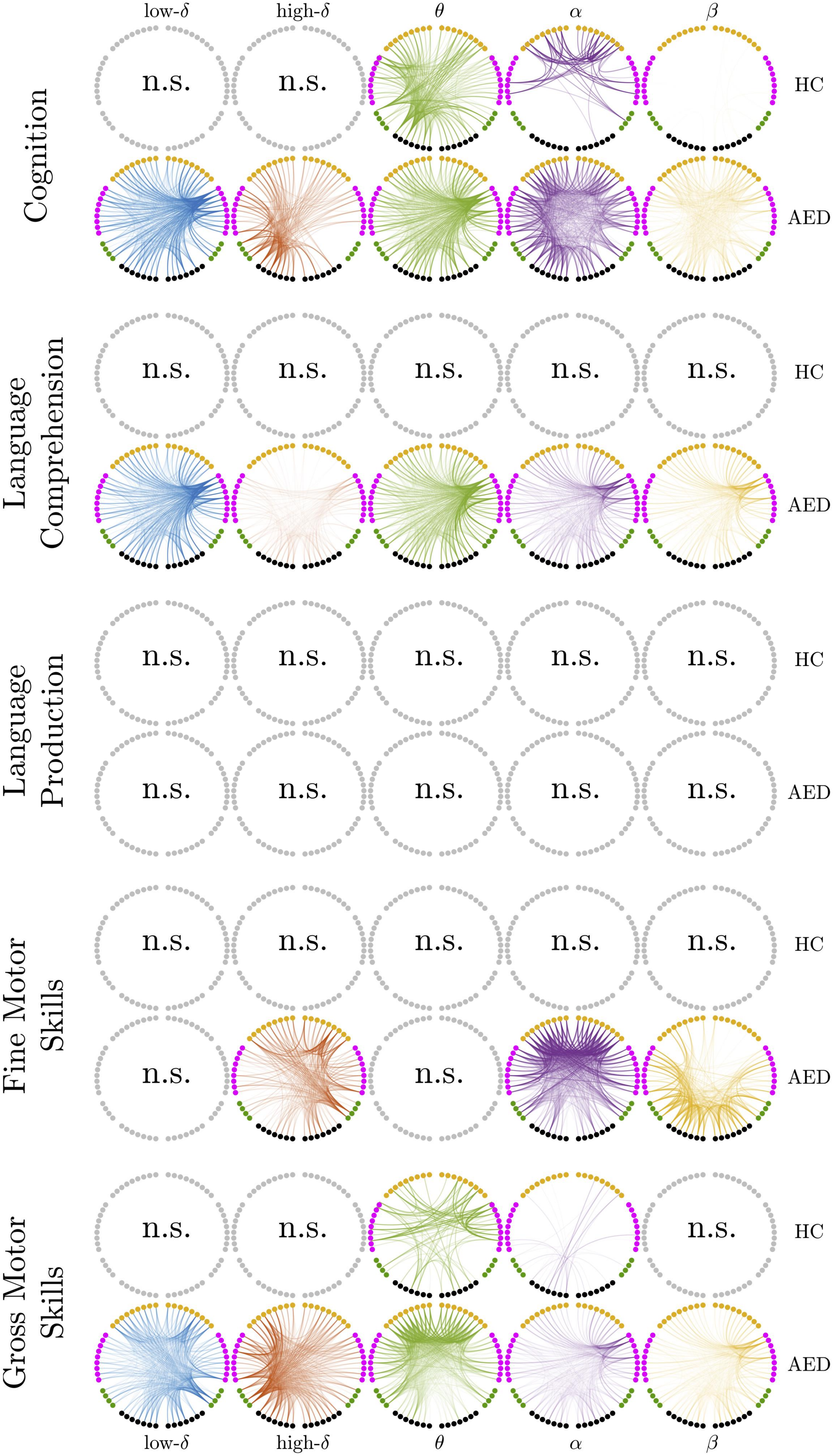

low-δ
high-δ
θ
α
β
Cognition
Language Comprehension
Language Production
Fine Motor Skills
Gross Motor Skills
HC
AED
n.s.

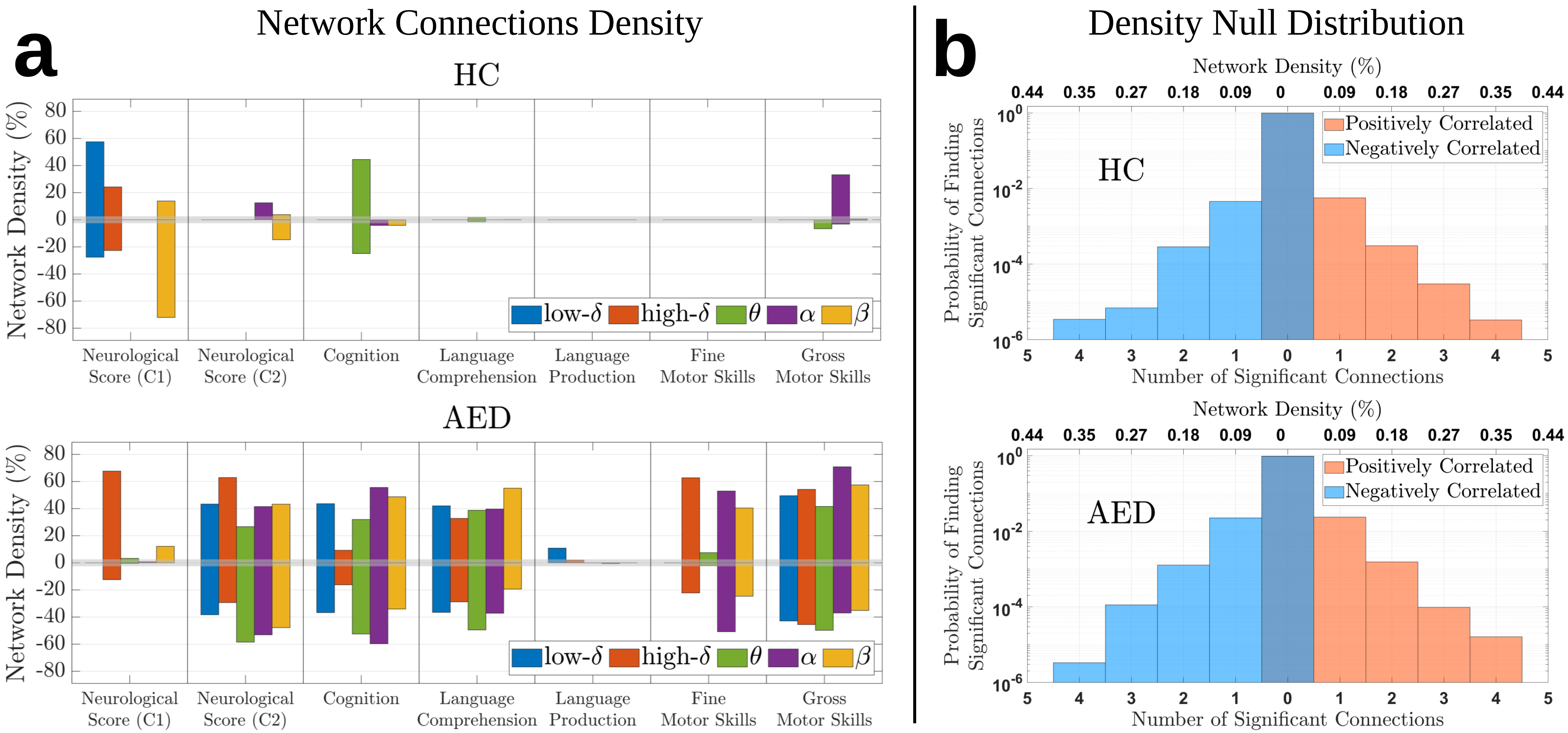

a
Network Connections Density
HC
Network Density (%)
80
60
40
20
0
-20
-40
-60
-80
low-δ
high-δ
θ
α
β
Neurological Score (C1)
Neurological Score (C2)
Cognition
Language Comprehension
Language Production
Fine Motor Skills
Gross Motor Skills
AED
b
Density Null Distribution
Network Density (%)
0.44
0.35
0.27
0.18
0.09
0
Probability of Finding Significant Connections
Positively Correlated
Negatively Correlated
Number of Significant Connections

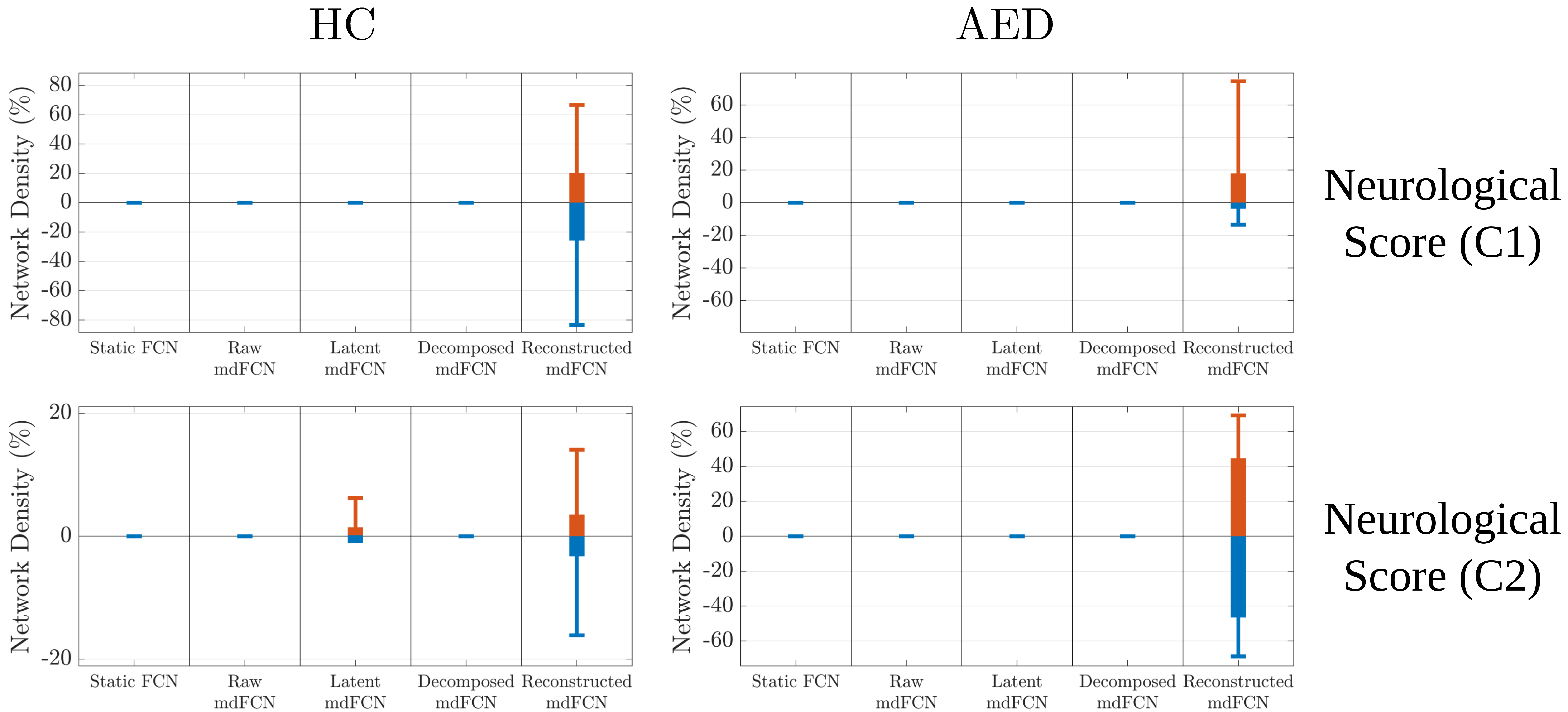
HC
AED
Network Density (%)
Static FCN
Raw mdFCN
Latent mdFCN
Decomposed mdFCN
Reconstructed mdFCN
Neurological Score (C1)
Neurological Score (C2)

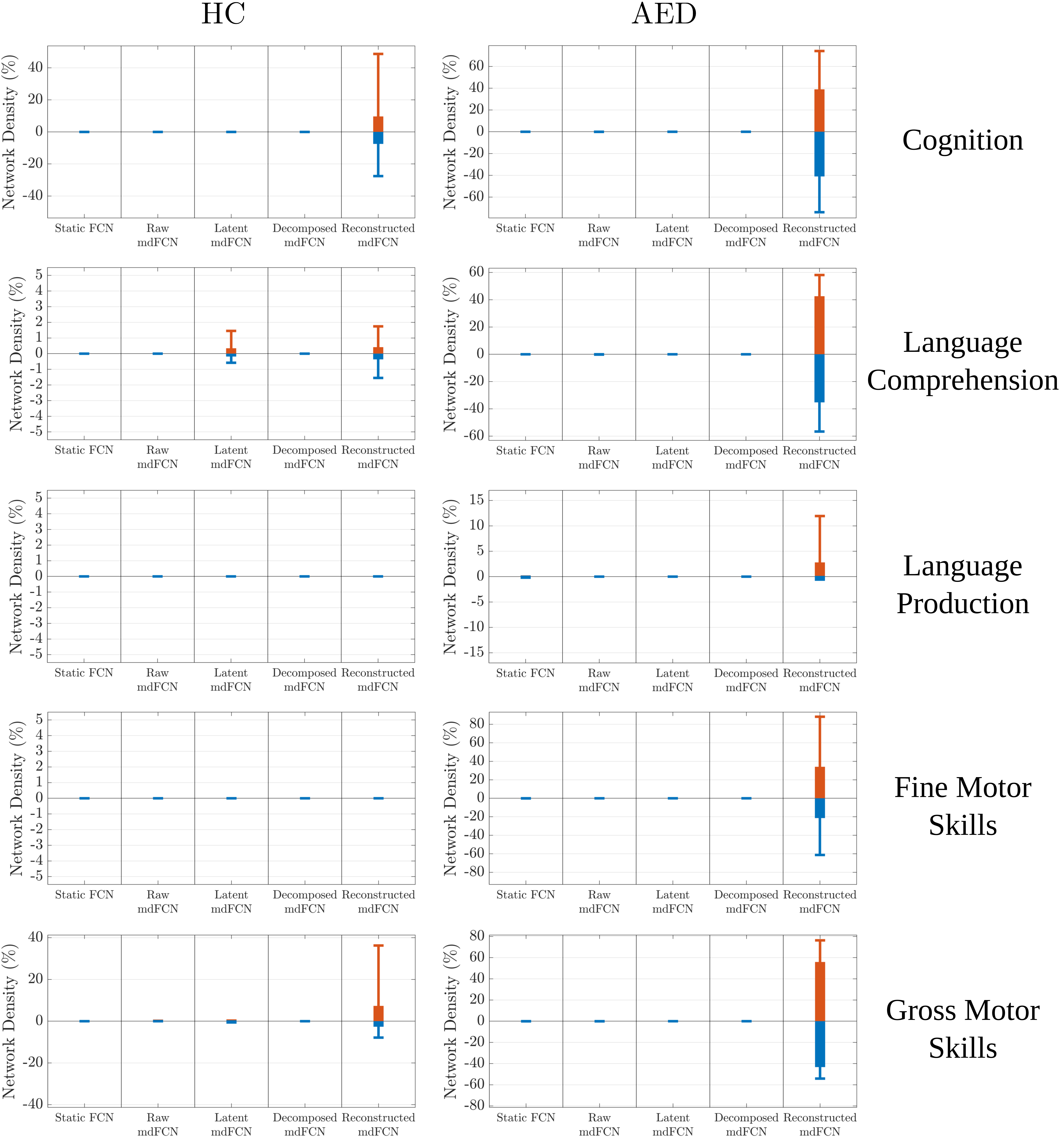
HC
AED
Cognition
Language Comprehension
Language Production
Fine Motor Skills
Gross Motor Skills
Network Density (%)
Static FCN
Raw mdFCN
Latent mdFCN
Decomposed mdFCN
Reconstructed mdFCN

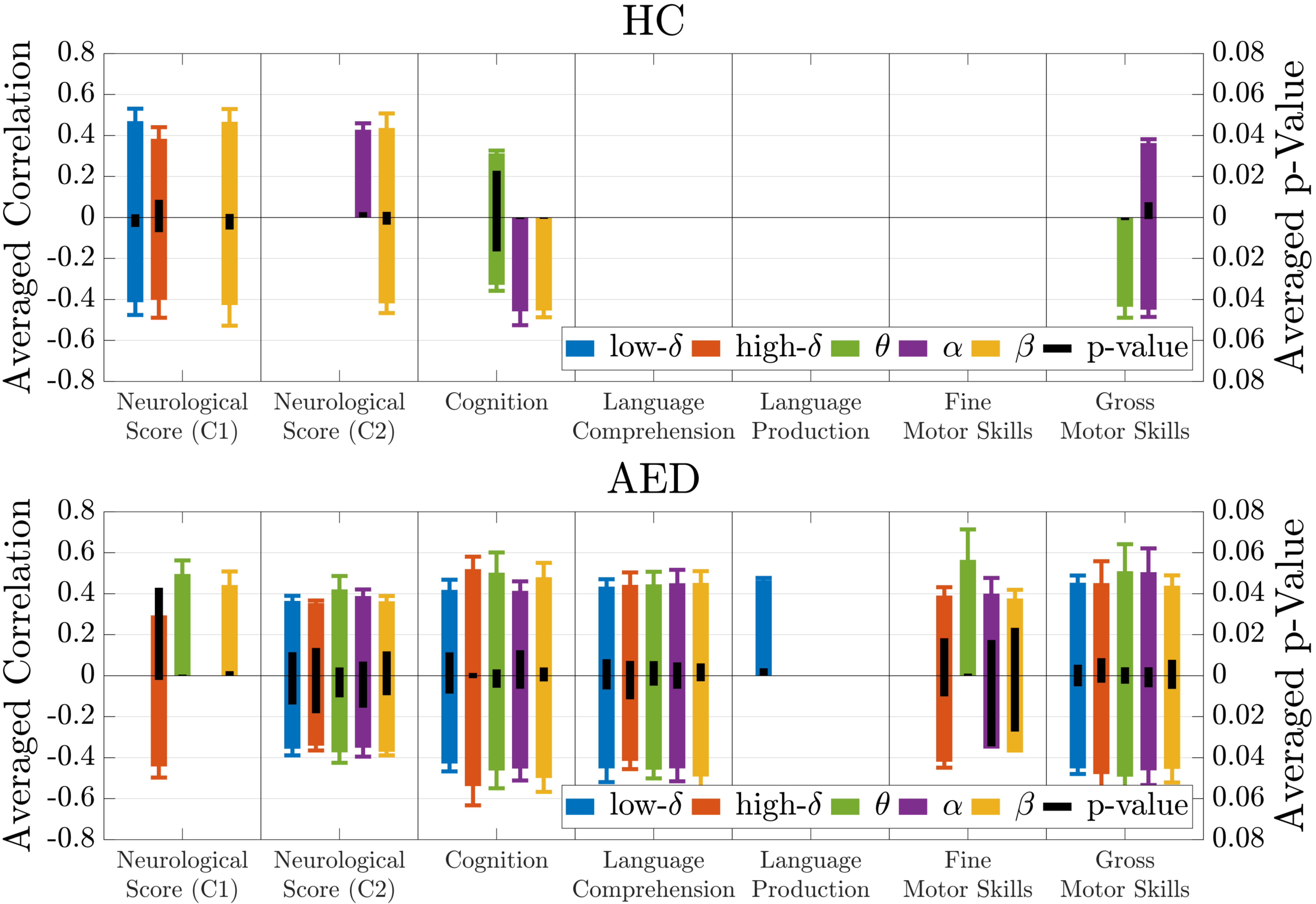

HC
AED
Averaged Correlation
Averaged p-Value
0.8
0.6
0.4
0.2
0
-0.2
-0.4
-0.6
-0.8
0.08
0.06
0.04
0.02
low-δ
high-δ
θ
α
β
p-value
Neurological Score (C1)
Neurological Score (C2)
Cognition
Language Comprehension
Language Production
Fine Motor Skills
Gross Motor Skills

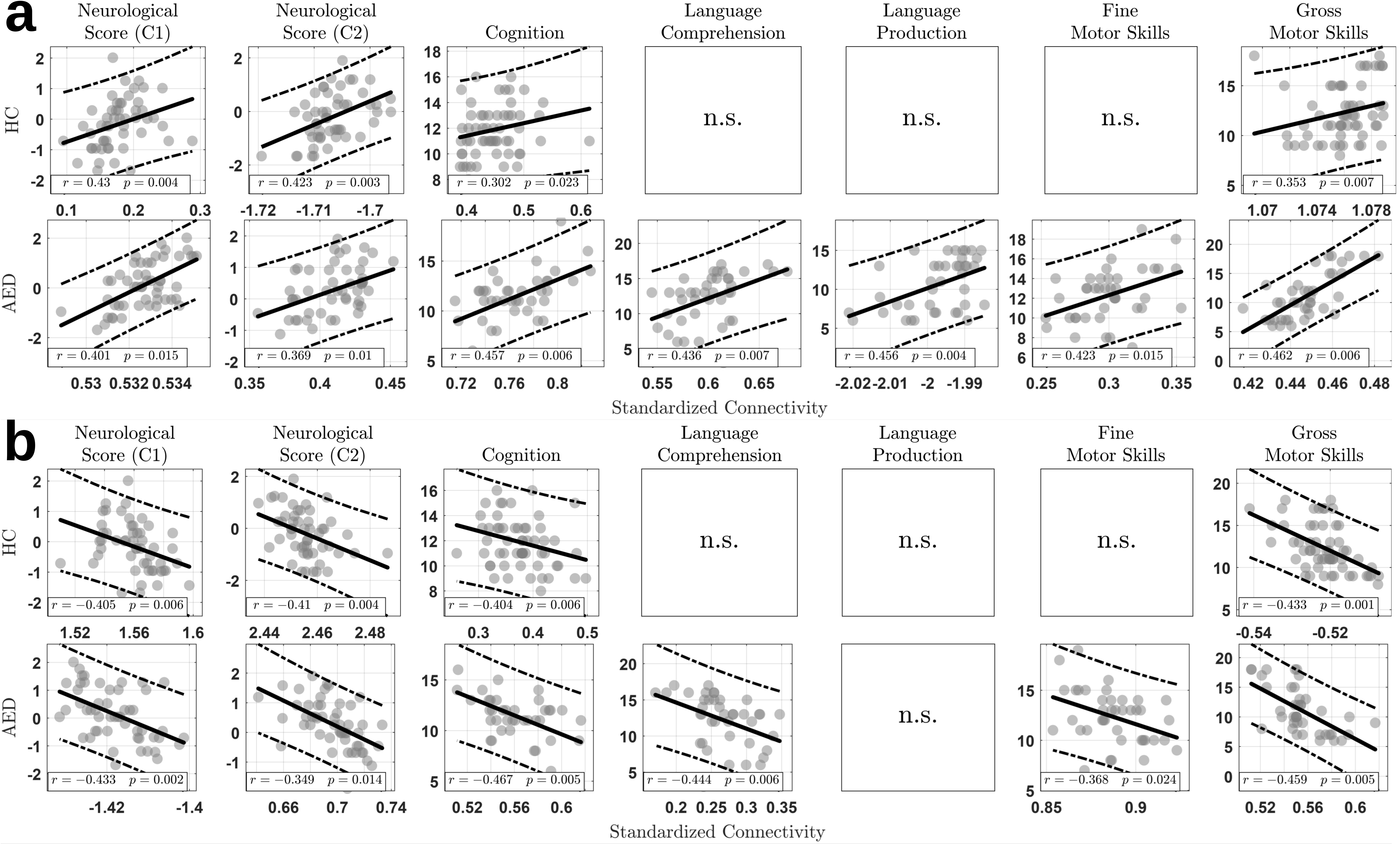
a
b
Neurological Score (C1)
Neurological Score (C2)
Cognition
Language Comprehension
Language Production
Fine Motor Skills
Gross Motor Skills
HC
AED
n.s.
$r = 0.43$ $p = 0.004$
$r = 0.423$ $p = 0.003$
$r = 0.302$ $p = 0.023$
$r = 0.353$ $p = 0.007$
$r = 0.401$ $p = 0.015$
$r = 0.369$ $p = 0.01$
$r = 0.457$ $p = 0.006$
$r = 0.436$ $p = 0.007$
$r = 0.456$ $p = 0.004$
$r = 0.423$ $p = 0.015$
$r = 0.462$ $p = 0.006$
$r = -0.405$ $p = 0.006$
$r = -0.41$ $p = 0.004$
$r = -0.404$ $p = 0.006$
$r = -0.433$ $p = 0.001$
$r = -0.433$ $p = 0.002$
$r = -0.349$ $p = 0.014$
$r = -0.467$ $p = 0.005$
$r = -0.444$ $p = 0.006$
$r = -0.368$ $p = 0.024$
$r = -0.459$ $p = 0.005$
Standardized Connectivity

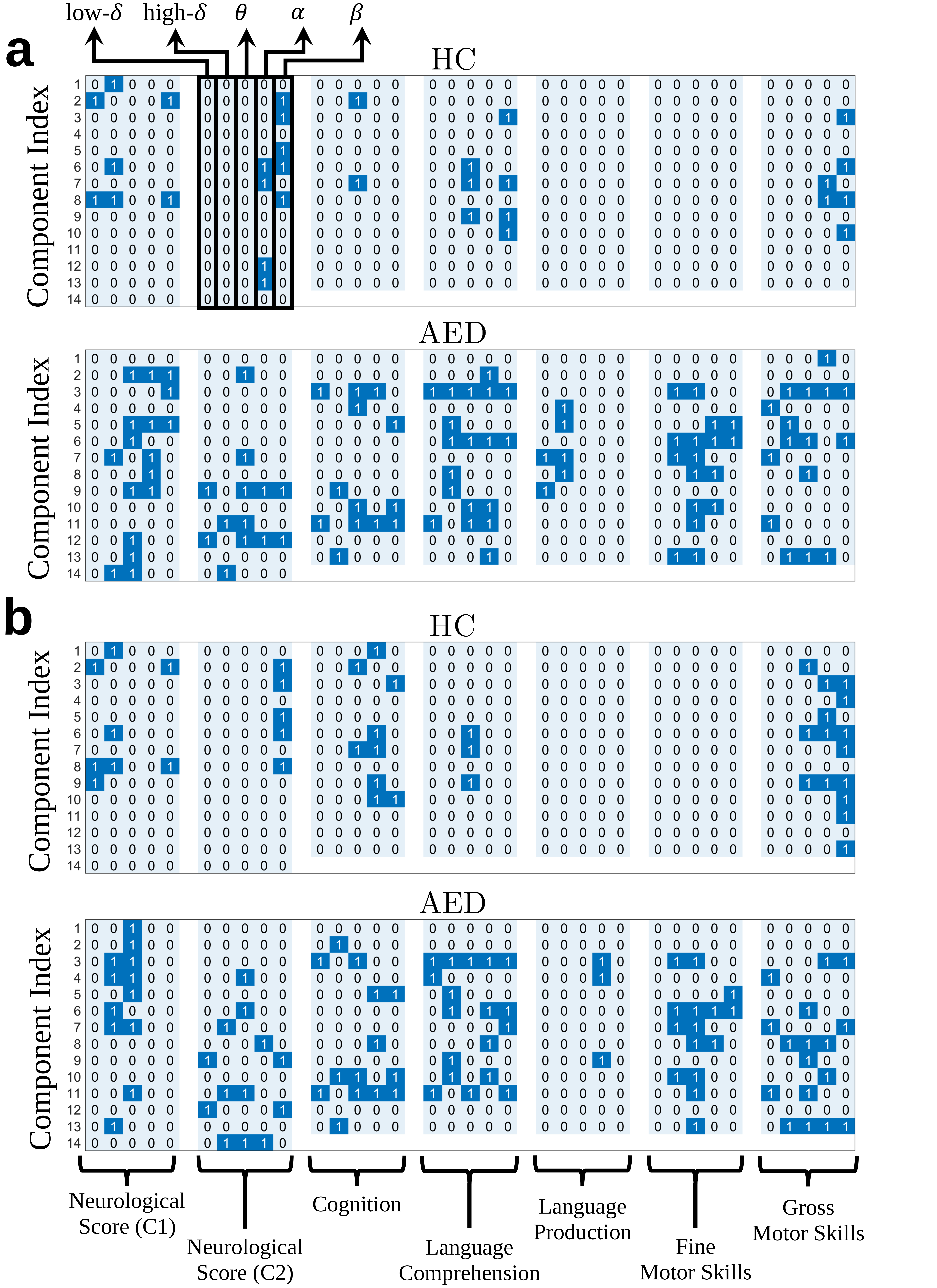

a
b
low-δ
high-δ
θ
α
β
HC
AED
Component Index
Neurological Score (C1)
Neurological Score (C2)
Cognition
Language Comprehension
Language Production
Fine Motor Skills
Gross Motor Skills
