# Supplementary material for: Multiplex dynamic networks in the newborn brain disclose latent links with neurobehavioral phenotypes
Source: Hum Brain Mapp. 2024 Feb 5;45(2):e26610. doi: 10.1002/hbm.26610 (PMC10839739; doi:10.1002/hbm.26610)
Supplement: Supplementary file 2 — DATA S2. Supporting Information. [file HBM-45-e26610-s002.docx]

Supplementary Material

This document supplements the main paper by presenting further details, mathematical formulations, and results.

# Individual dynamic functional connectivity

The time-varying phase-phase coupling between pairs of cortical signals, measured by the debiased weighted phase lag index, is formulated as follows:

$$x_{i,j,k}=\left| \frac{\left( \int\mathfrak{I}\left( \mathcal{S}_{i,j,k}(f) \right)df \right)^{2}-\int\mathfrak{I}\left( \mathcal{S}_{i,j,k}\left( f \right) \right)^{2}df}{\left( \int\left| \mathfrak{I}\left( \mathcal{S}_{i,j,k}(f) \right) \right|df \right)^{2}-\int\mathfrak{I}\left( \mathcal{S}_{i,j,k}\left( f \right) \right)^{2}df} \right| ,$$

where $x_{i,j,k}$ denotes the strength of cortico-cortical synchronization between regions $i$ and *j* in a time window $k$, $\mathcal{S}_{i,j,k}\left( f \right)$ is the complex-valued cross-spectra of the cortical signals in the window $k$, and $\mathfrak{I(\cdot)}$ denotes the imaginary part. By repeating this calculation for different frequency bands, a 3-way connectivity tensor corresponding to pairwise connectivity, time windows, and frequency bands was generated for each individual infant as described in Fig. 1.


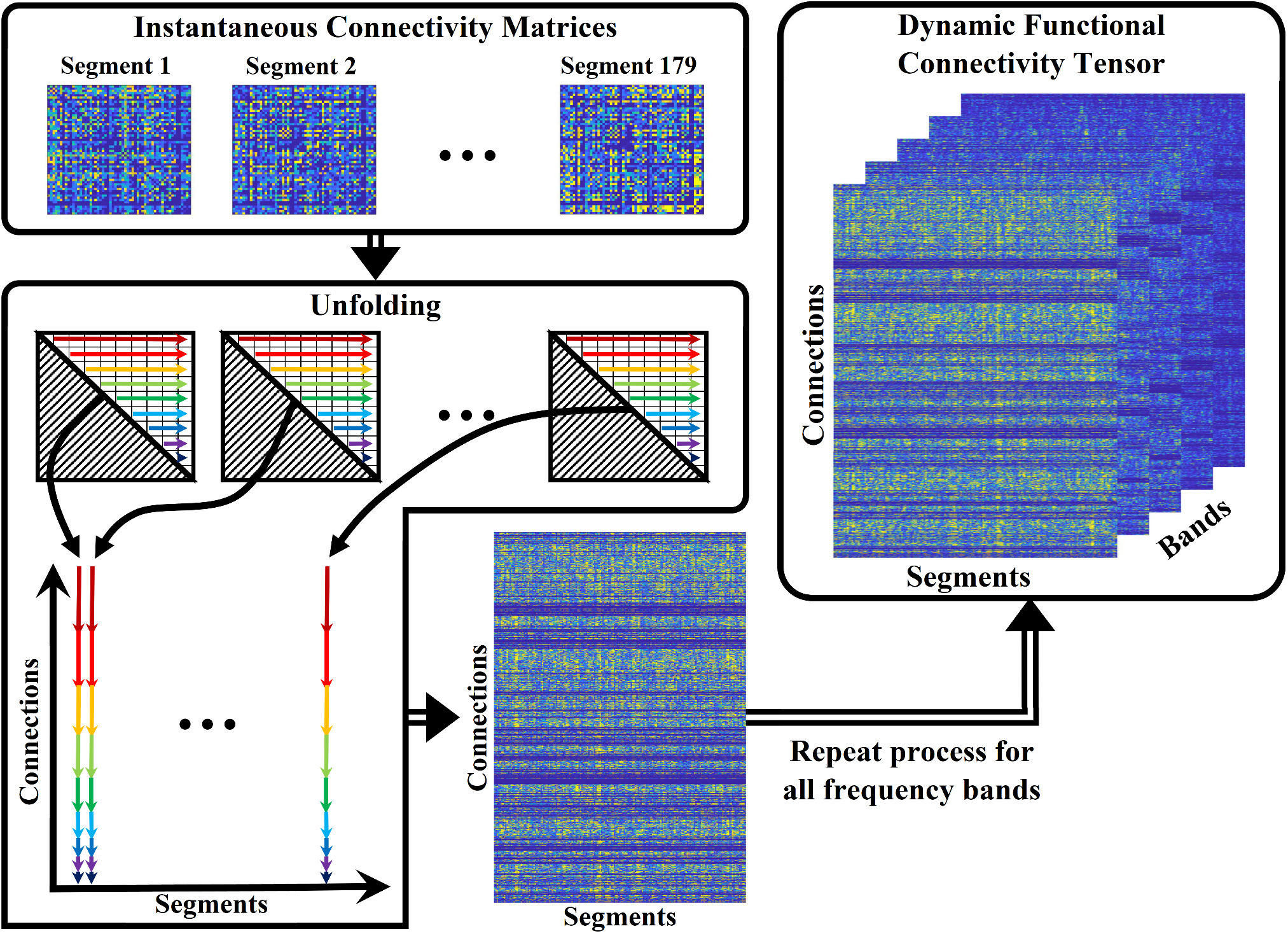


Fig. 1 The 3-way connectivity tensor for an individual subject. The process is based on vectorizing the upper triangle of the instantaneous connectivity matrix of every time window to become a column in a matrix describing connectivity across pairwise connections and segments. Matrices corresponding to different frequency bands are then arranged as frontal slices in a 3-way tensor describing connectivity across pairwise connections, segments, and frequency bands.

# Non-negative matrix factorization

Let $\mathbf{X}=[X_{1},X_{2},\cdots,X_{5}]$ be the group-level mdFCN tensor with $X_{i}$ being the frequency slice $i$. We applied the NMF to each frontal slice in $\mathbf{X}$ independently and the resultant estimates were aggregated back to the original tensor form as follows:

$$X_{i}\approx\hat{X}_{i} = W_{i} H_{i} ,$$

where $\hat{X}_{i}$ is the NMF estimated frontal slice of frequency band $i$, $W_{i}$ and $H_{i}$ are non-negative matrices of sizes 1128×*P* and *P*×179*N*, respectively, *P* is the NMF model order which reflects the estimation’s complexity, and $\hat{\mathbf{X}}=\left[ \hat{X}_{1},\hat{X}_{2},\cdots,\hat{X}_{5} \right]$ is the extracted group-level latent mdFCN. We solved for the NMF factor matrices ($W_{i}$ and $H_{i}$) by minimizing the estimation squared error using the alternating non-negativity constrained least squares algorithm with the following settings: the maximum number of iterations was 10^3^, the fitting residual threshold was 10^-4^, the estimation error threshold was 10^-4^, and the factor matrices were initialized randomly from a positive uniform distribution.

# Canonical polyadic decomposition

The CPD defines $\hat{\mathbf{X}}$ as the sum of $Q$ tensors with each being the outer product of three non-negative vectors *a*, *b*, and *c*, i.e.:

$$\hat{\mathbf{X}}\approx\sum_{q=1}^{Q} a_{q}\otimes b_{q}\otimes c_{q} ,$$

where $Q$ is the number of decomposed components which reflects the approximation’s intricacy, $\otimes$ denotes the outer product of vectors, and $a_{q}$, $b_{q}$, and $c_{q}$ are the pairwise connection, segment/subject, and spectral factors of sizes 1128×1, 179*N*×1, and 5×1, respectively. We solved for the CPD factors by minimizing the estimation squared error using the alternating least-squares algorithm with the following settings: the maximum number of iterations was 10^3^, the fitting residual threshold was 10^-8^, the estimation error threshold was 10^-8^, and the CPD factors were initialized randomly from a positive uniform distribution.

# Entropy-based model selection technique

## The NMF model order

We designed an automatic model order selection technique based on entropy to avoid under and over-fitting problems when estimating $\hat{\mathbf{X}}$. In the context of this work, under-fitting results in undescriptive group-level features while over-fitting carries inter-subject differences and noise. Therefore, a balance between the two is needed to find the group’s common global structure. The developed method relies on the fact that by increasing the model’s order, the estimate’s complexity becomes higher to account for the input’s variability. In other words, by minimizing the NMF estimation error, the output’s entropy increases and converges to the input’s original level. The main advantage of using entropy instead of error for measuring the model’s adequacy is its ability to reflect the amount of explained information rather than variance. In addition, it resolves the model selection uncertainty when dealing with approximate linearly declining estimation errors (see Fig. 2). Consequently, entropy offers a better indication for the sufficiency of the model to generate informative and relevant group-level latent networks. The NMF model order selection strategy is summarized as follows:

1. Given an NMF order $P\in[1, 40]$, solve for $\hat{X}_{i}$ and calculate the normalized estimation entropy, i.e.: $ℇ_{i}\left( P \right)=E(\hat{X}_{i})/E(X_{i})$ where $E(X_{i})$ is the Shannon entropy of $X_{i}$.
2. Compute the normalized estimation entropy rate of change, i.e.: $ℇ_{i}^{'}\left( P \right)=ℇ_{i}\left( P \right)-ℇ_{i}\left( P-1 \right)$.
3. Repeat steps 1-2 to account for the random initialization and average the rate of change $\overline{ℇ_{i}^{'}}(P)$.
4. Define a threshold $\epsilon$ that is above the lowest value in $\overline{ℇ_{i}^{'}}(P)$ by 5% of its range and find the best order $P_{i}^{*}$ as follows:

$$P_{i}^{*}=\left\lfloor\underset{P}{\mathrm{argmax}} \left( \overline{ℇ_{i}^{'}}(P) \right) \right\rfloor+1 s.t. \overline{ℇ_{i}^{'}}\leq\epsilon.$$

We repeated this procedure for every frequency band of interest $i$ yielding $P^{*}=\left[ P_{1}^{*},P_{2}^{*},\cdots,P_{5}^{*} \right]$, and 10 times for every estimate and model order to account for the random initialization.


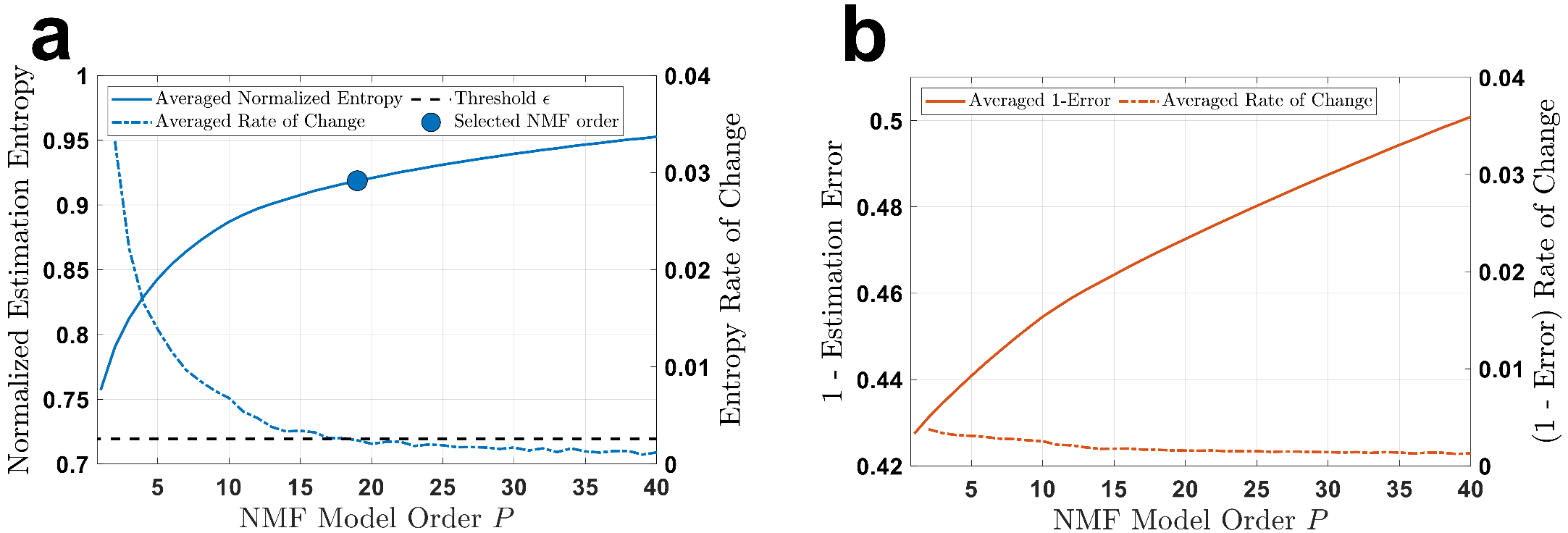


Fig. 2 Comparing entropy and error for selecting an NMF model order. a Entropy reflects the amount of explained information with respect to the model’s order and demonstrates a converging behavior that facilitates selecting an adequate model order. b Error, or more precisely 1-error, communicates the amount of explained variance and shows a linear trend that convolutes model order selection; it is difficult to favor any order. We utilized the healthy group-level tensor for this comparison, and plotted Entropy/1-Error (left y-axes) along with their rates of change (right y-axes) in solid and dashed lines, respectively.

## The CPD number of components

We extended the entropy-based technique introduced in Section ‎4.1 for tensors to select the CPD best number of components $Q^{*}$, i.e.:

1. Given a number of components $Q\in[1, 40]$, decompose $\hat{\mathbf{X}}$ into $Q$ components and calculate the normalized estimation entropy $ℇ(Q)$.
2. Compute the normalized estimation entropy rate of change $ℇ^{'}(Q)$.
3. Repeat steps 1-2 to account for the random initialization and average the rate of change $\overline{ℇ^{'}}(Q)$.
4. Define a threshold $\epsilon$ that is above the lowest value in $\overline{ℇ^{'}}(Q)$ by 5% of its range and find the best order $Q^{*}$ as follows:

$$Q^{*}=\left\lfloor\underset{Q}{\mathrm{argmax}} \left( \overline{ℇ^{'}}(Q) \right) \right\rfloor+1 s.t. \overline{ℇ^{'}}\leq\epsilon.$$

This procedure suppresses any remaining inter-subject variability and noise, but it is mainly intended to breakdown the latent mdFCNs into subnetworks of manageable size for further analysis. Besides, it was repeated 20 times for every estimate and model order to account for the random initialization. Finally, when comparing the decomposed mdFCNs of two clinical groups, the number of components was unified to omit any artificial group-differences; it was set to the maximum of the two estimates.

# Multiplex functional connectivity networks

## Formulation

A mathematical tensor representation $\mathbf{Z}$ can be transformed to an equivalent multiplex weighted network $\boldsymbol{W}=\left\{ W^{\left[ 1 \right]},W^{\left[ 2 \right]}, \cdots,W^{\left[ M_{L} \right]} \right\}$ consisting of $M_{L}$ active layers, $M_{E}$ edges, and $M_{D}$ nodes with $W^{\left[ m \right]}=\left\{ w_{ij}^{\left[ m \right]} \right\}$ where $w_{ij}^{\left[ m \right]}=z_{ijm}$ and $z_{ijm}$ is an element in $\mathbf{Z}$ denoting the connectivity between regions $i$ and $j$ at the frequency band $m$ (see Fig. 3 to visualize this transformation). Besides, an unweighted network $\boldsymbol{A}=\left\{ A^{\left[ 1 \right]},A^{\left[ 2 \right]}, \cdots,A^{\left[ M_{L} \right]} \right\}$ can be formed to summarize the edges’ state in a simpler way with $A^{\left[ m \right]}=\left\{ a_{ij}^{\left[ m \right]} \right\}$ where $a_{ij}^{\left[ m \right]}=1$ if $w_{ij}^{\left[ m \right]}>0$, $a_{ij}^{\left[ m \right]}=0$ otherwise.


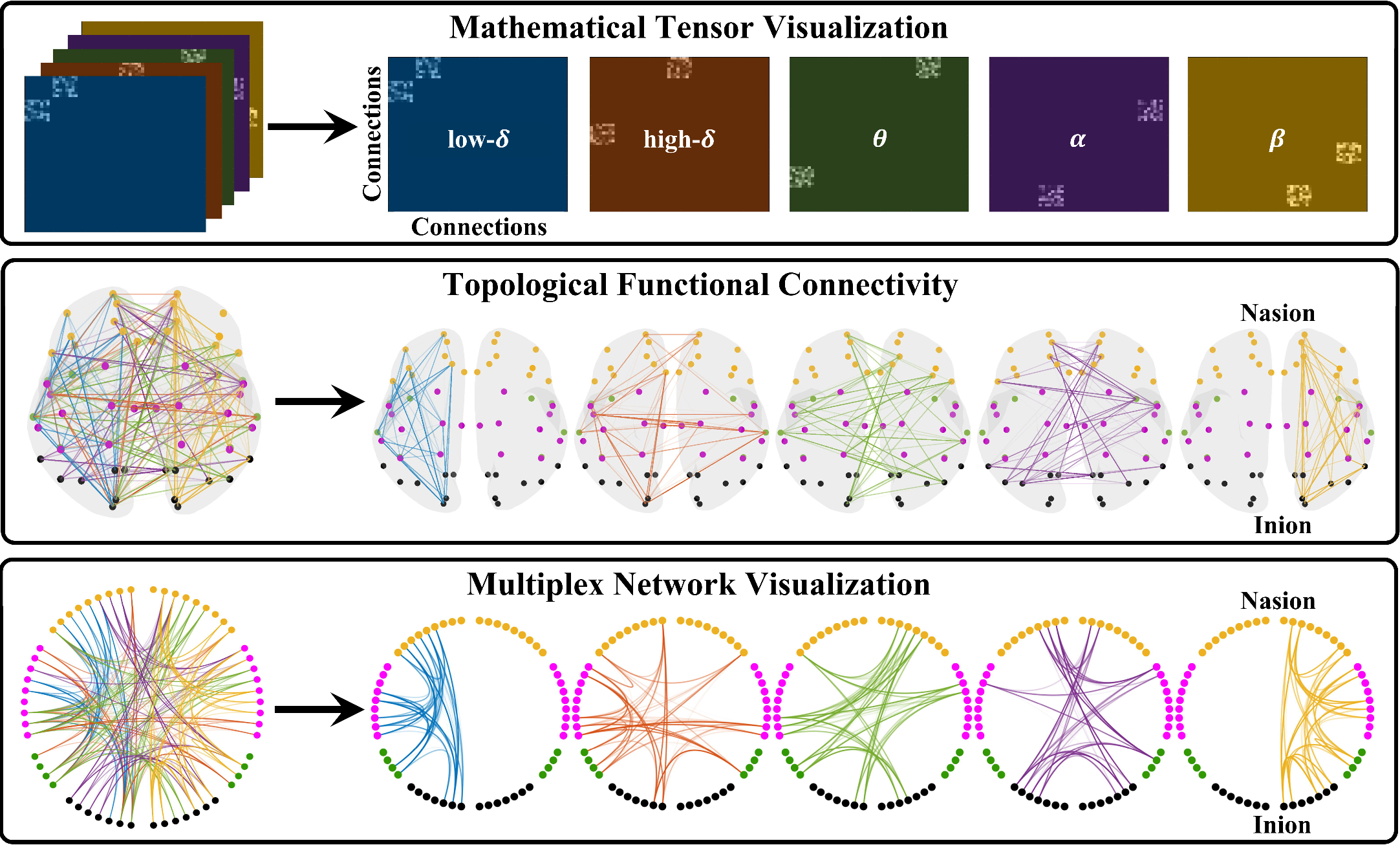


Fig. 3 The 3-way mathematical tensor presented by three visualizations that focus on different aspects and structural patterns. The multiplex network visualization uses a connectogram to associate the cortical regions on its nodes, coming from the topological functional connectivity, with their weighted/unweighted links.

## Node structure

A node $i$ is considered active on layer $m$ if it is connected to at least another node such that $k_{i}^{[m]}>0$ where $k_{i}^{[m]}=\sum_{j=1}^{M_{D}} a_{ij}^{\left[ m \right]}$ is the total number of active connections of node $i$ on layer $m$. The normalized overlapping degree $\mathcal{O}_{i}$ of a node $i$ represents its overall relevance in terms of the relative number of incident edges and it is defined as follows:

$$\mathcal{O}_{i}=\frac{1}{M_{L}\left( M_{D}-1 \right)}\sum_{m=1}^{M_{L}} k_{i}^{[m]} .$$

Moreover, the multiplex participation coefficient $\mathcal{P}_{i}$ describes the distribution of incident edges across the layers and it is defined as:

$$\mathcal{P}_{i}=\frac{M_{L}}{M_{L}-1}\left( 1-\sum_{m=1}^{M_{L}} \left( \frac{k_{i}^{[m]}}{\sum_{m=1}^{M_{L}} k_{i}^{[m]}} \right)^{2} \right), s.t. M_{L}>1 .$$

The pair of node measures $\left( \mathcal{P}_{i},\mathcal{O}_{i} \right)$ encodes the multiplex cartography of the node $i$. On the one hand, multiplex, mixed, and focused nodes are defined by the following participation levels: $\mathcal{P}_{i}>2/3$, $1/3\leq\mathcal{P}_{i}\leq2/3$, and $\mathcal{P}_{i}<1/3$, respectively. On the other hand, hubs and leaves are defined by the skewness of the overlapping degree distribution such that: negative/positive or right/left skewness indicates a tendency towards hubs/leaves.

## Layer structure

The similarity between two layers $m_{1}$ and $m_{2}$ is measured by the pairwise multiplexity $\mathcal{S}$ which computes the relative number of nodes that are active on both layers, i.e.:

$$\mathcal{S}^{[m_{1},m_{2}]}=\frac{1}{M_{D}}\sum_{i=1}^{M_{D}} b_{i}^{[m_{1}]}b_{i}^{[m_{2}]} ,$$

where $b_{i}^{\left[ m \right]}=1$ if $k_{i}^{[m]}>0$ and $b_{i}^{\left[ m \right]}=0$ otherwise, $\mathcal{S}^{[m_{1},m_{2}]}=1$ when all nodes are active in both layers, $\mathcal{S}^{[m_{1},m_{2}]}=0$ when no node is simultaneously active on the layers. In addition, the similarity between two layers can also be measured by the Hamming distance, i.e.:

$$\mathcal{H}^{[m_{1},m_{2}]}=\frac{\sum_{i=1}^{M_{D}} b_{i}^{[m_{1}]}\left( 1-b_{i}^{[m_{2}]} \right)+b_{i}^{[m_{2}]}\left( 1-b_{i}^{[m_{1}]} \right)}{\min\left( \sum_{i=1}^{M_{D}} b_{i}^{[m_{1}]}+b_{i}^{[m_{2}]},M_{D} \right)} ,$$

where $\mathcal{H}$ is small for similar layer activities and large for dissimilar ones.

## Edge structure

The pair of nodes $\left( i,j \right)$ in the network can be connected through many edges with a total overlap ratio $\mathcal{O}_{e}$ defined by:

$$\mathcal{O}_{e}=\frac{1}{M_{L}M_{E}}\sum_{m=1}^{M_{L}} \sum_{i=1}^{M_{D}} k_{i}^{[m]} .$$

Furthermore, the overall edge intersection index $\mathcal{X}$ measures the probability of finding a pair of nodes that is connected by an edge on all layers as follows:

$$\mathcal{X=}M_{L}\left( \frac{\sum_{i=1}^{M_{D}} \sum_{j=1}^{M_{D}} \min\left( \left[ a_{ij}^{\left[ 1 \right]}, a_{ij}^{\left[ 3 \right]},\cdots,a_{ij}^{\left[ M_{L} \right]} \right] \right)}{\sum_{m=1}^{M_{L}} \sum_{i=1}^{M_{D}} k_{i}^{[m]}} \right) , s.t. M_{L}>1 .$$

# Supplementary results

This section presents the paper’s additional and supplementary results.


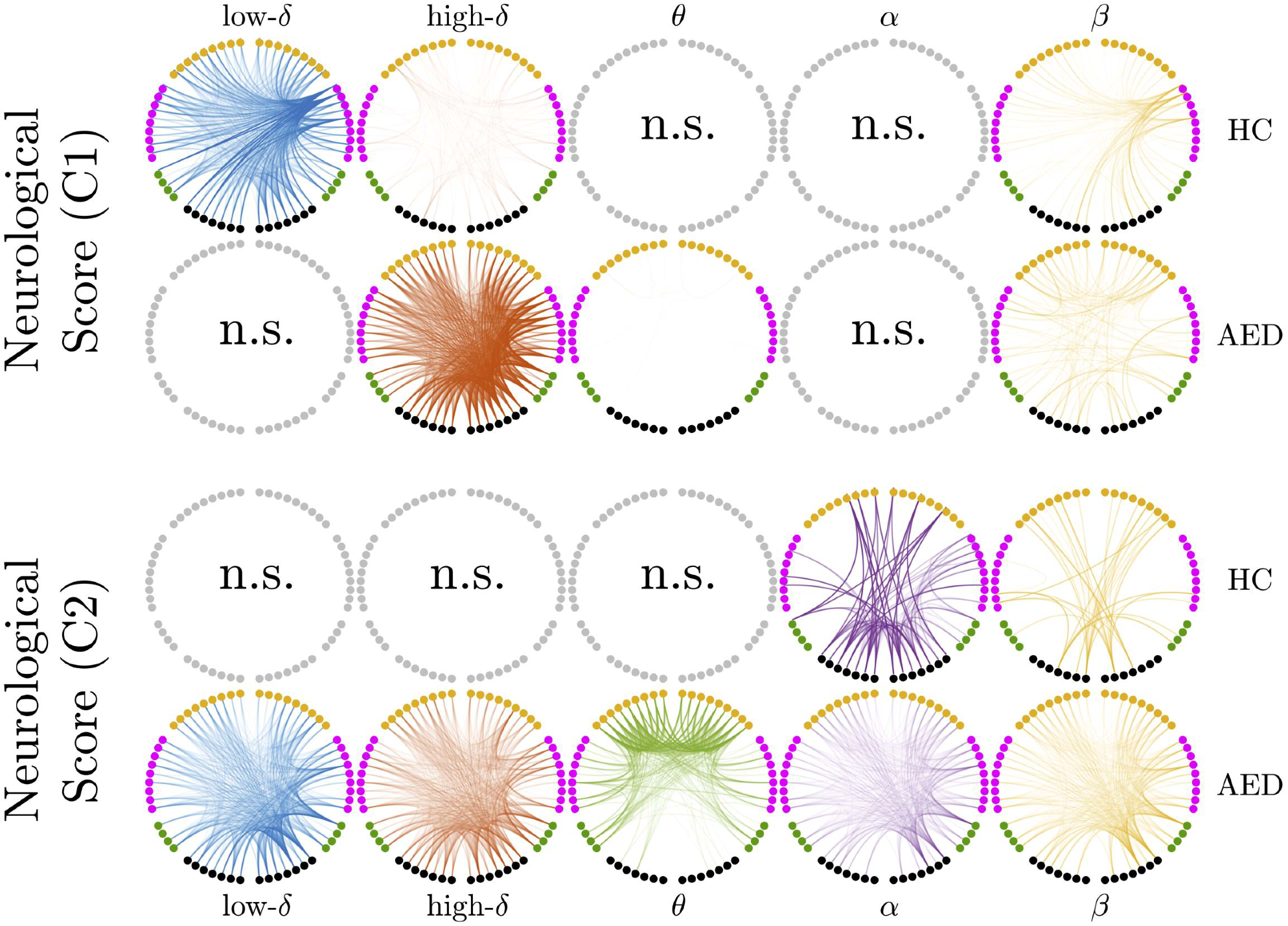


Fig. 4 The mdFCN positive correlates to the short-term neurological performance. The brain connections are demonstrated for every frequency band of interest, their color transparency is enhanced to ease visualization by squaring the connectivity values, and n.s. denotes non-significant results.


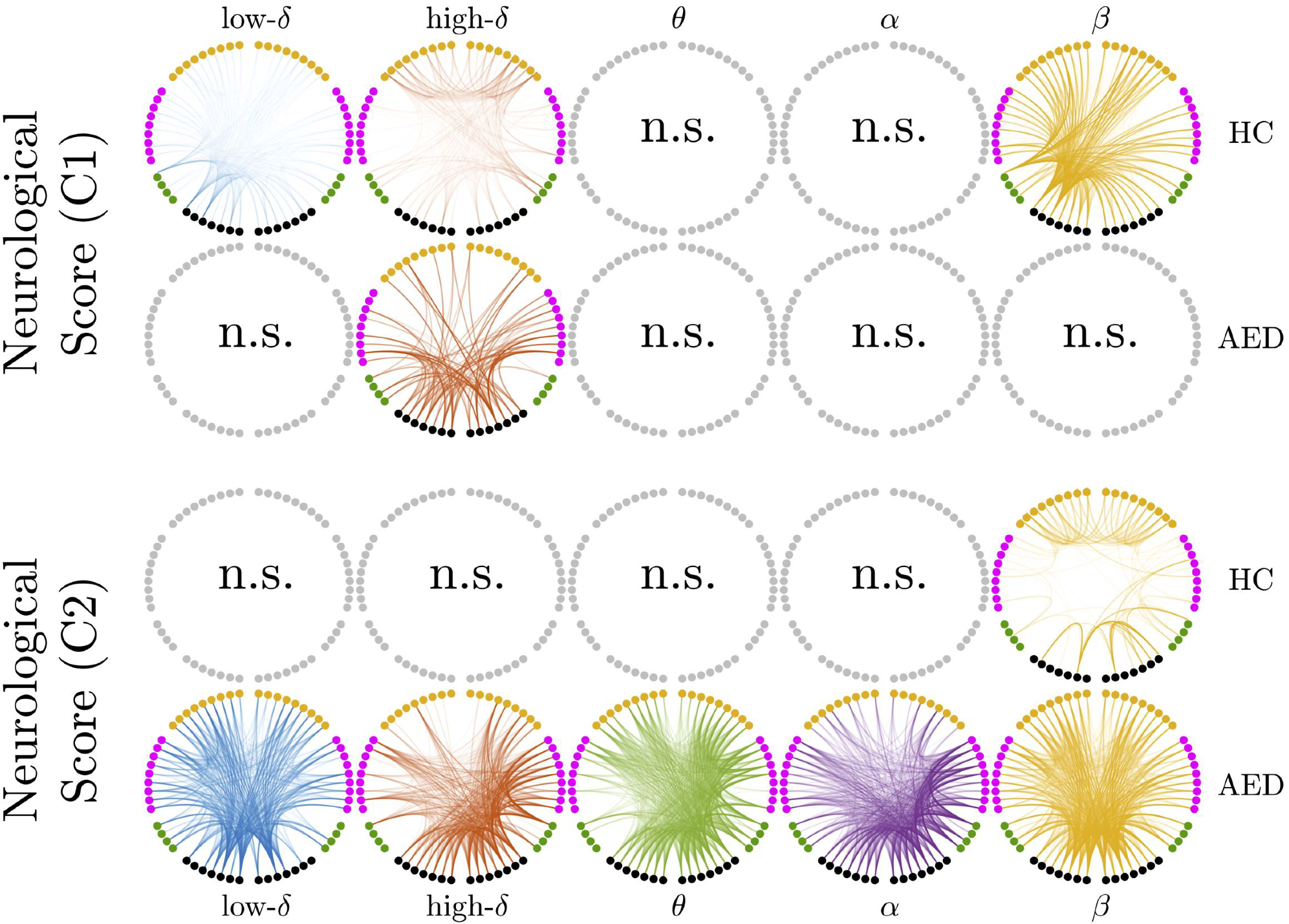


Fig. 5 The mdFCN negative correlates to the short-term neurological performance. The brain connections are demonstrated for every frequency band of interest, their color transparency is enhanced to ease visualization by squaring the connectivity values, and n.s. denotes non-significant results.


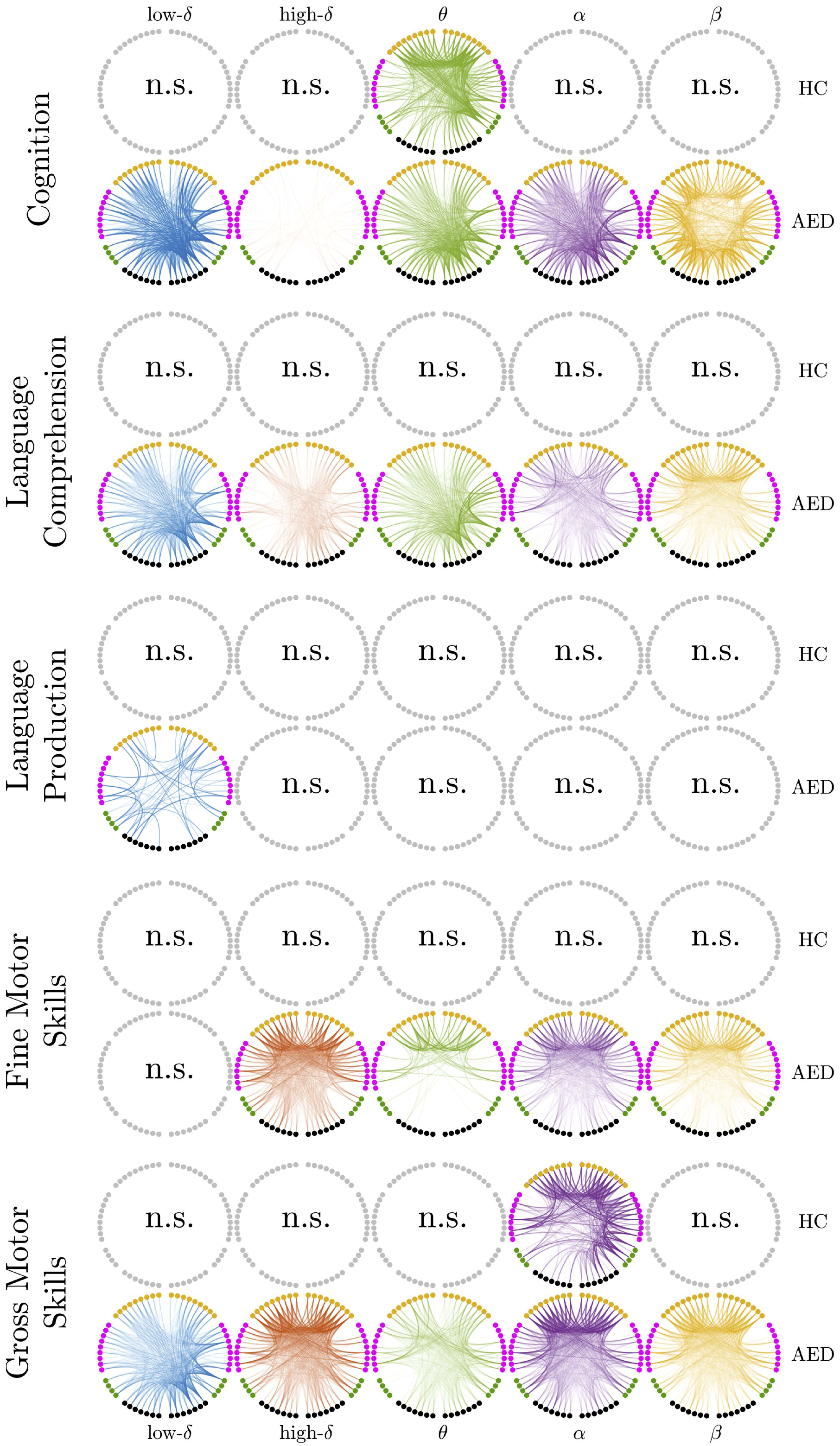


Fig. 6 The mdFCN positive correlates to the long-term developmental outcomes. The brain connections are demonstrated for every frequency band of interest, their color transparency is enhanced to ease visualization by squaring the connectivity values, and n.s. denotes non-significant results.


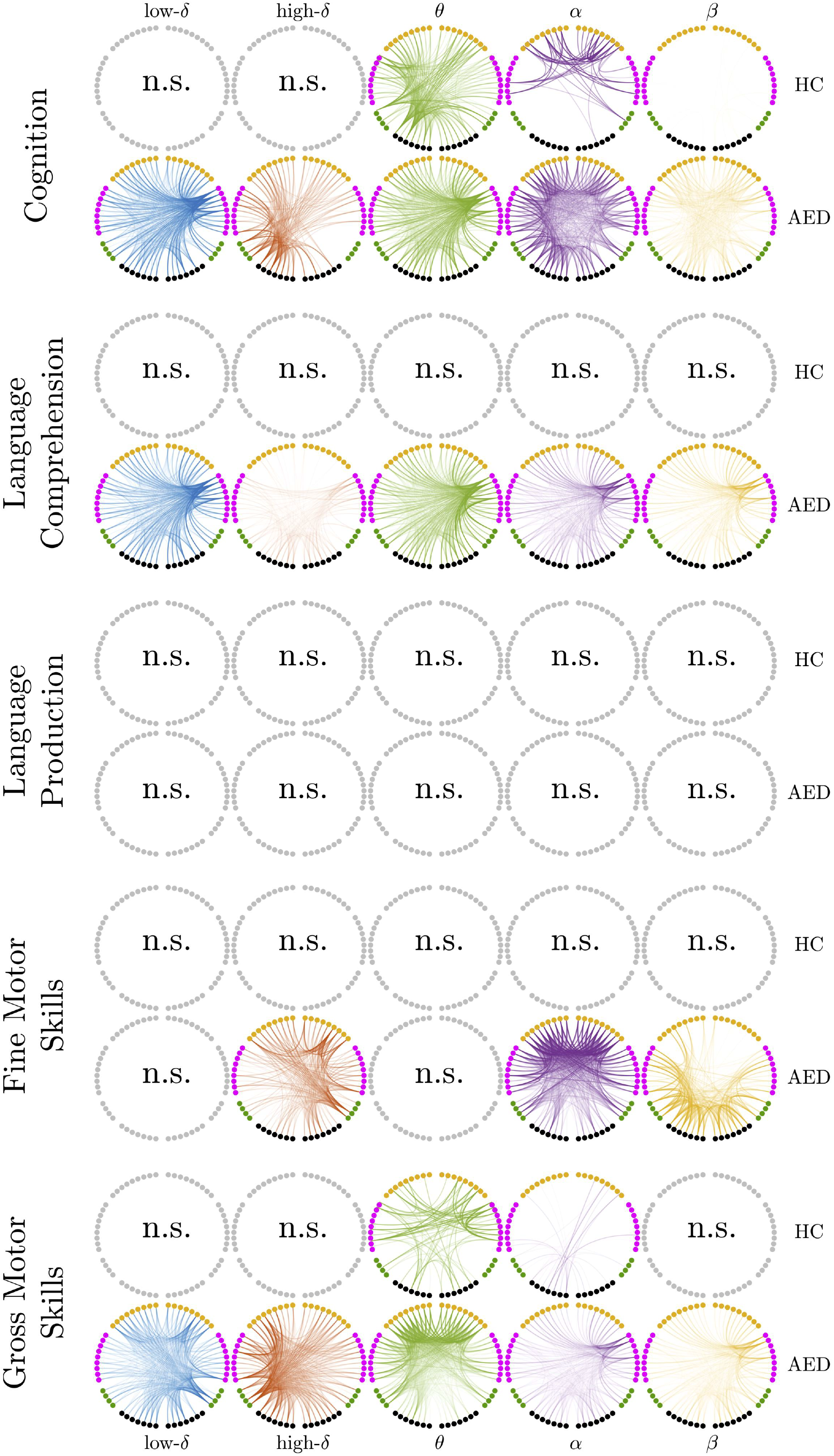


Fig. 7 The mdFCN negative correlates to the long-term developmental outcomes. The brain connections are demonstrated for every frequency band of interest, their color transparency is enhanced to ease visualization by squaring the connectivity values, and n.s. denotes non-significant results.


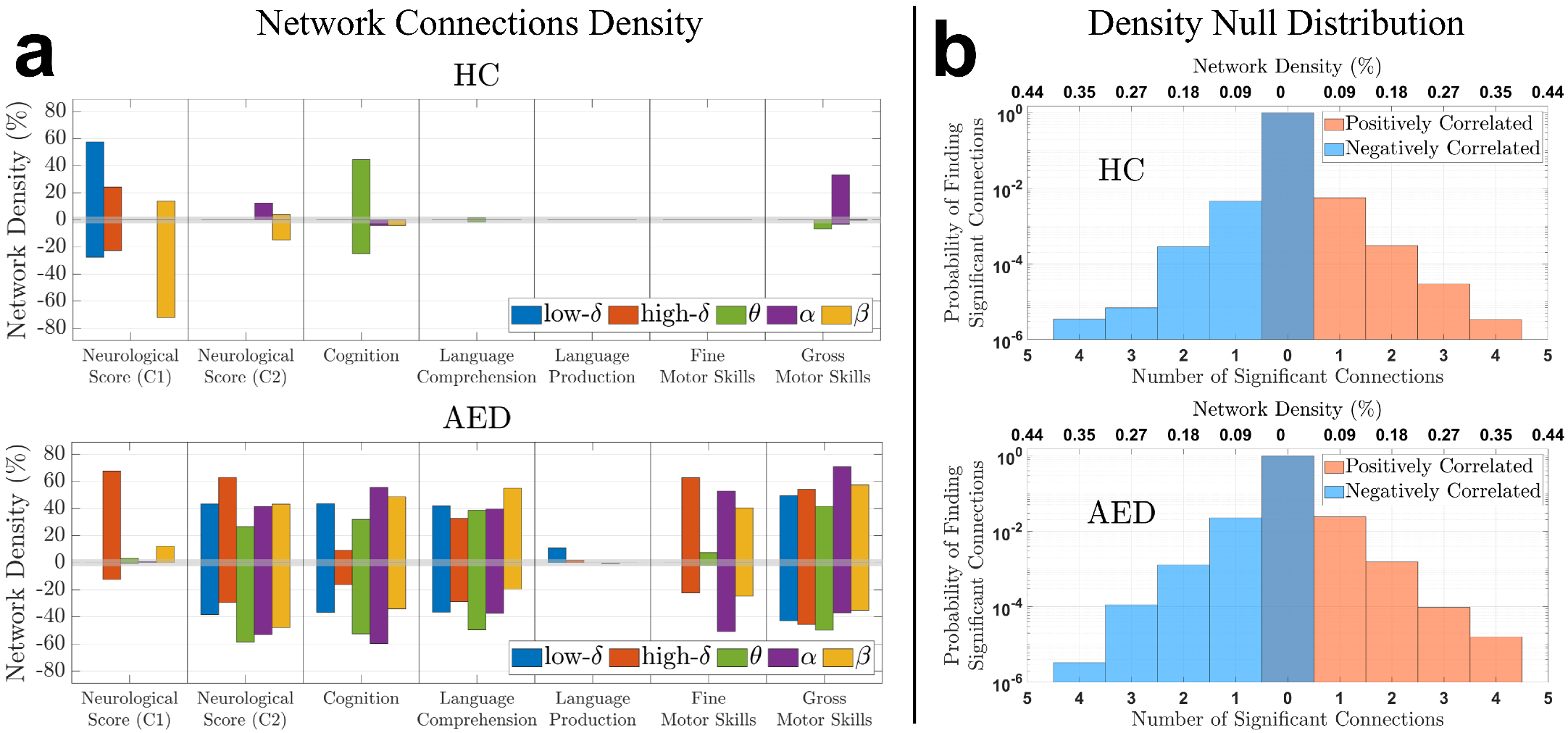


Fig. 8 The mdFCN relative number of connections that significantly correlate with phenotypes. a The network densities for both clinical groups and every frequency band of interest with positive/negative densities representing the relative number of connections yielding significant positive/negative correlations with the neurobehavioral phenotypes. Densities below the 2.5% mark, shown in gray, are discarded from analysis. b The network density null distribution for both clinical groups and correlation directions. The null distributions were generated by permuting the infants’ connections 10,000 times; calculating the number of connections yielding significant correlations at every permutation; and lastly, by computing the densities’ frequency. Note that the y-axes in c are shown on a logarithmic scale to ease visualization and interpretation.


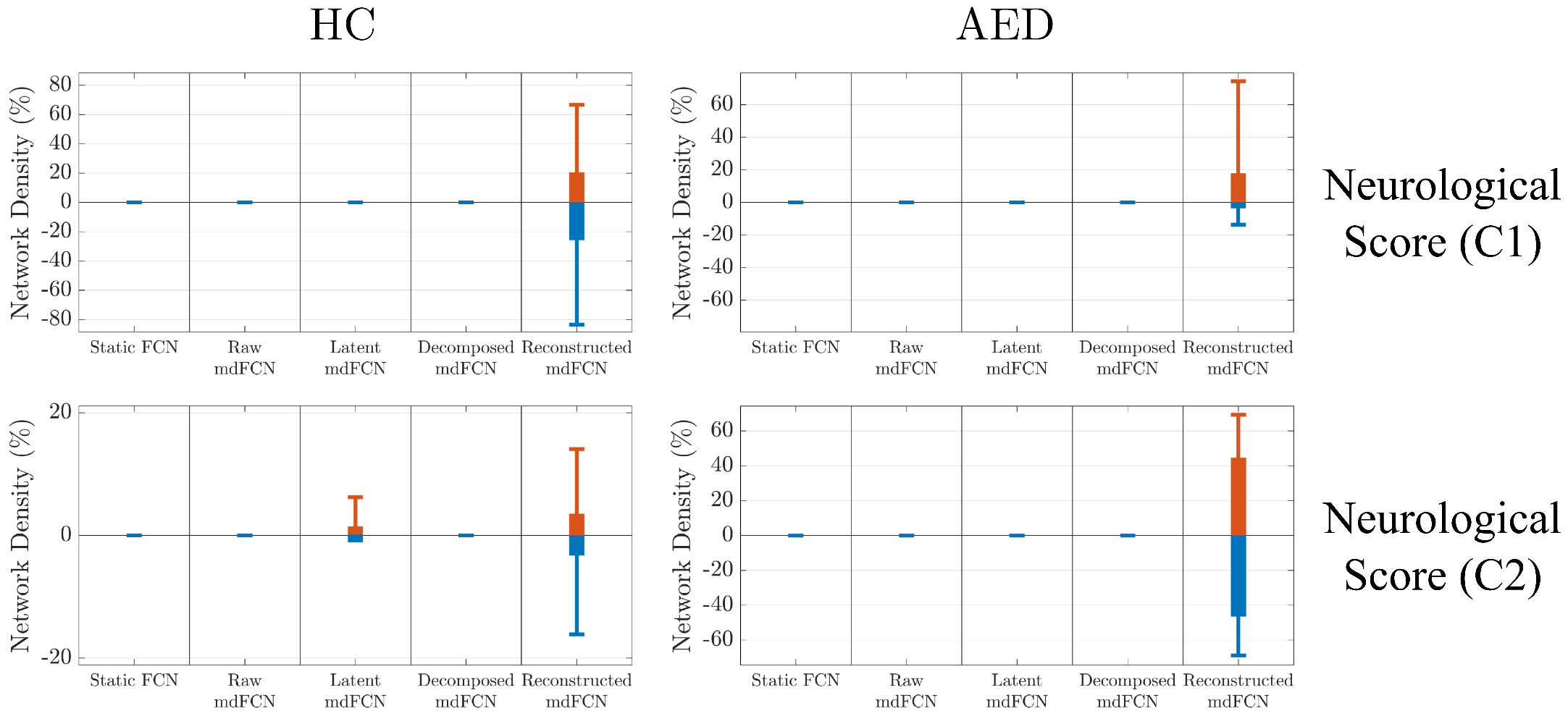


Fig. 9 The mdFCN verification using the short-term neurological scores. The results show that each stage in the proposed pipeline is essential to achieve local goals that, when combined, allows for uncovering latent network properties that correlate with the short-term clinical scores.


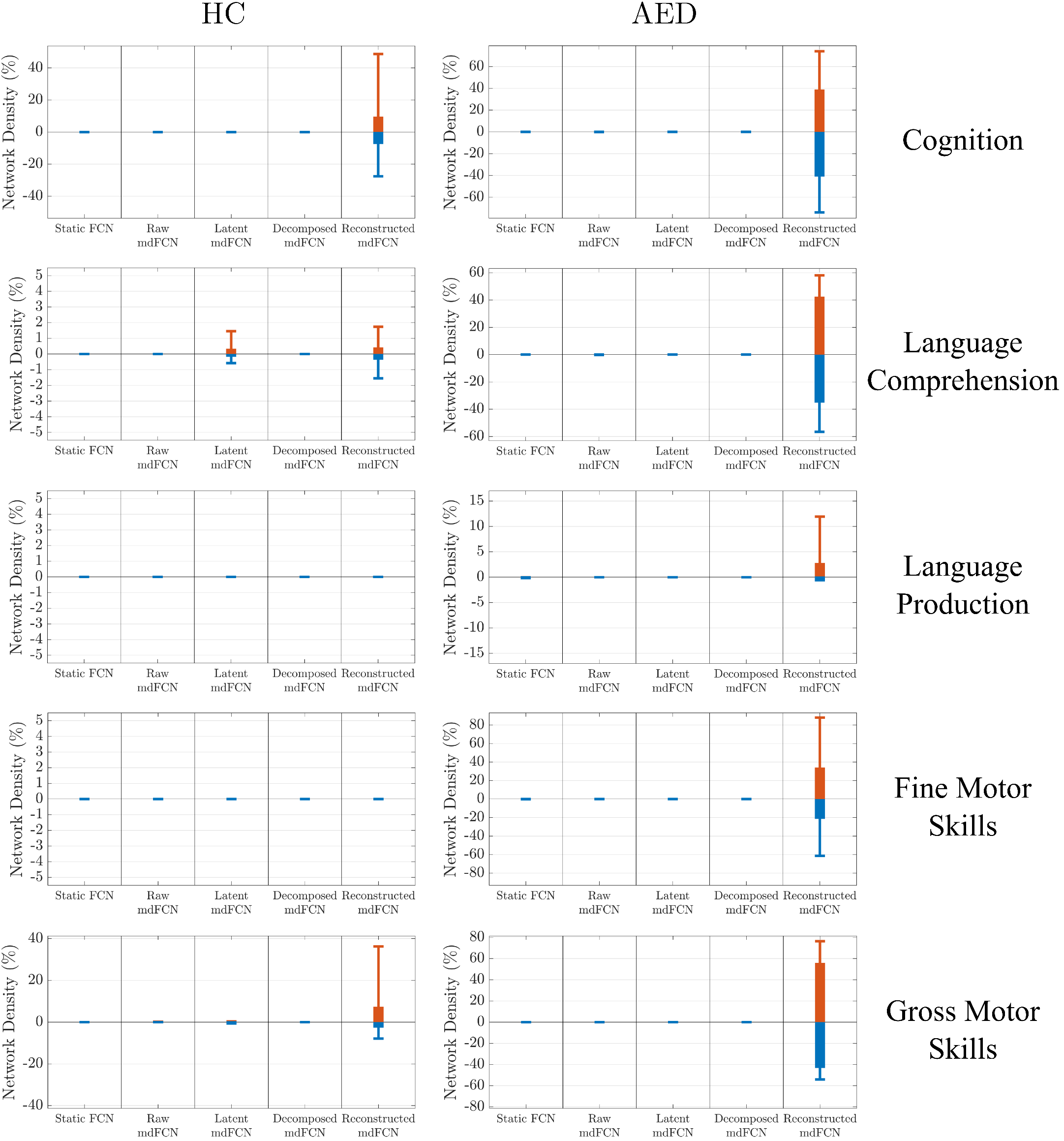


Figure 10 The mdFCN verification using the long-term developmental scores. The results show that each stage in the proposed pipeline is essential to achieve local goals that, when combined, allows for uncovering latent network properties that correlate with the long-term clinical scores.


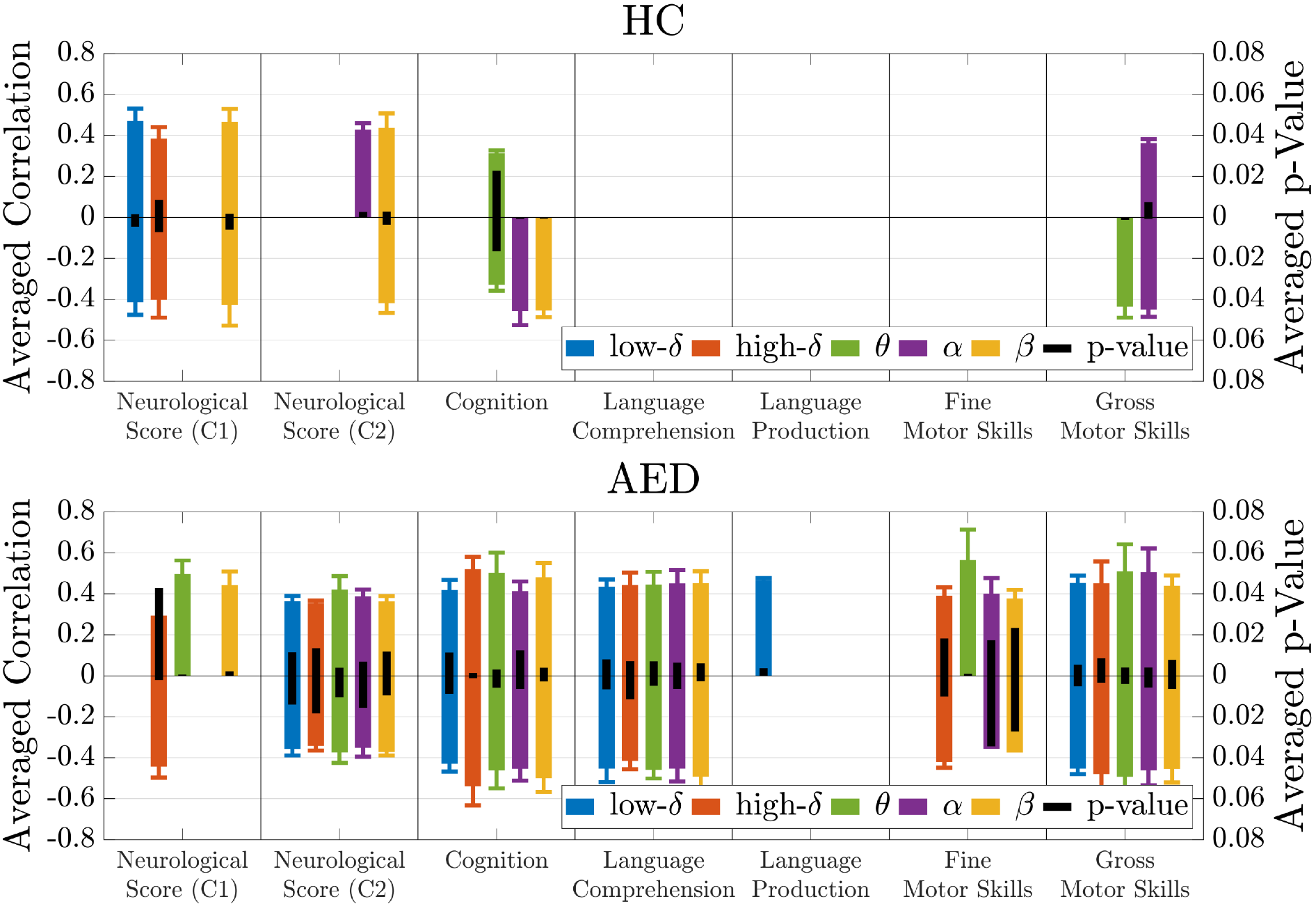


Fig. 11 The mdFCN correlations. The averaged positive/negative correlation with the neurobehavioral scores in every frequency band along with its p-value (in black) are explained by the left and right y-axes, respectively.


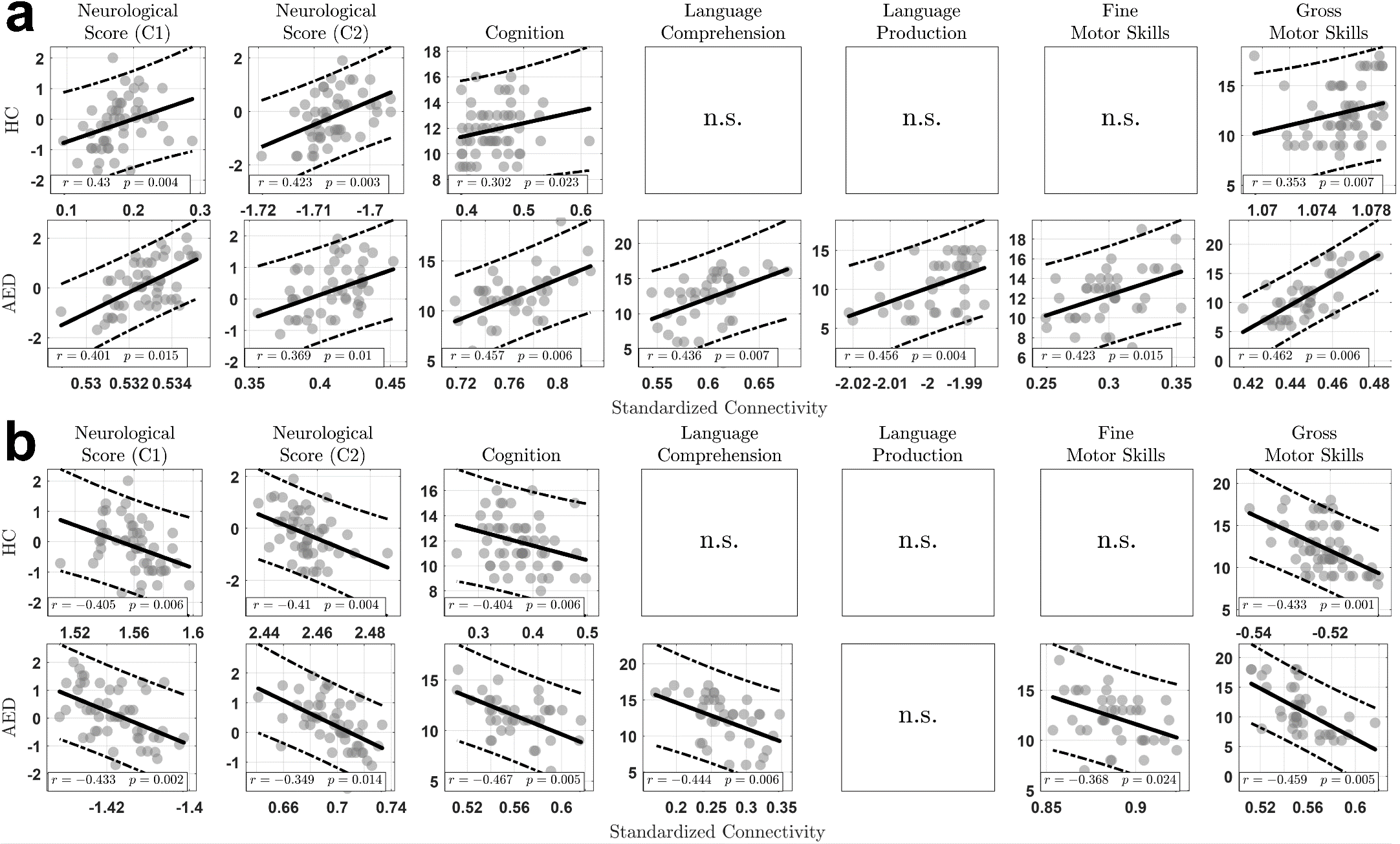


Fig. 12 The mdFCN predictive capacity. a The averaged standardized connectivity that positively correlates to phenotypes. b The averaged standardized connectivity that negatively correlates to phenotypes. The linear regression outputs are shown in solid lines along with their 95% confidence intervals in dashed lines and n.s. denotes non-significant results.


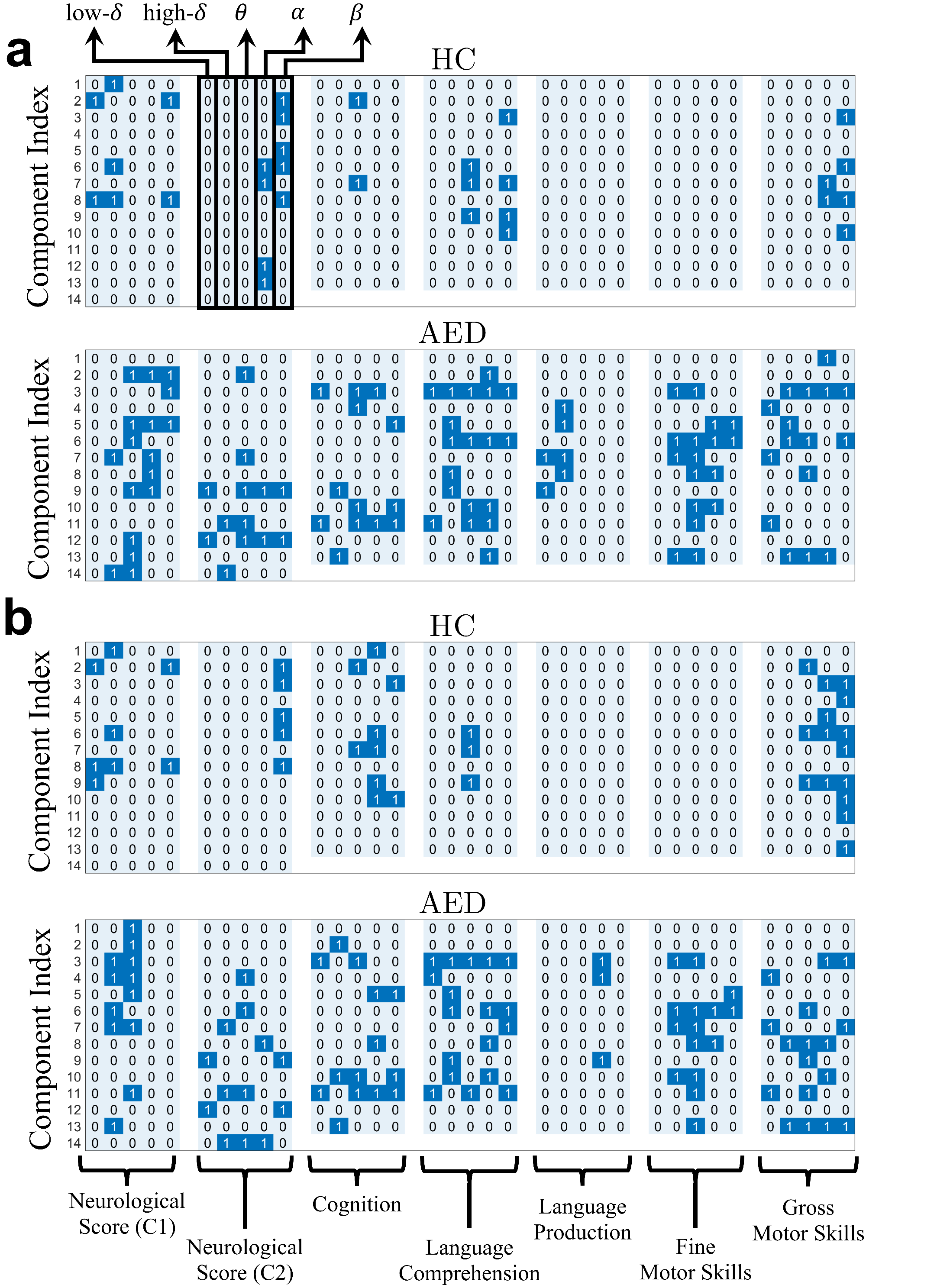


Fig. 13 The best network reconstruction strategies. a The set of selected components for positively correlated networks. b The set of selected components for negatively correlated networks. The results are shown for the HC and AED groups, for every neurobehavioral score, and for the five frequency bands of interest.
